# What are 40 Acres and a Mule Worth in the Twenty-First Century? A Rapid Review of Modern Reparative Initiatives to Address Socioeconomic and Health Inequality Among Black Americans

**DOI:** 10.1007/s40615-025-02513-7

**Published:** 2025-06-30

**Authors:** Jocelyn L. Brown

**Affiliations:** Ohio University, 6 President St #305, Athens, OH 45701 USA

**Keywords:** Reparations, Black Americans, Socioeconomic inequality, Health disparities, American Descendants of Slavery

## Abstract

The historical call for reparations for Black Americans has recently regained relevance. Reparations aim to address not only generational inequality established during slavery but also the perpetuation of socioeconomic and health disparities over time via Jim Crow, redlining, police brutality, and other forms of structural and systemic racism. Using Public Health Critical Race Praxis (PHCRP), this rapid review assesses the landscape of reparative initiatives across the United States at the governmental and organizational levels, analyzing the eligibility criteria, political ideologies, and goals of various stakeholders. Additionally, it examines the potential ability of reparations initiatives to reduce socioeconomic and health inequality. Key themes identified include American Descendants of Slavery (ADOS) should be eligible for reparations; the overall goals of reparative initiatives differ between governments and organizations; reparations extend far beyond direct payments; and reparations initiatives are influenced by one another but largely uncoordinated. The ways in which these themes align with PHCRP and the extensive policy implications are discussed.

## Background

The call for reparations for Black Americans began before the end of chattel slavery in 1865 and has increased in intensity in recent years [1–3]. Reparations refer to measures implemented to rectify social, political, and economic harm inflicted on individuals or groups [4]. In addition to roughly 250 years of chattel slavery, civil rights activists have cited Jim Crow segregation, discriminatory mortgage and business loan lending practices such as redlining, police brutality, and even the disproportional impact of COVID-19 on Black communities as grounds for reparations [3, 5–8].

Black Americans, 60% of whom are American Descendants of Slavery (ADOS), have been subjected to racism at the structural, systemic, institutional, and interpersonal levels [9, 10]. Structural racism takes the form of values such as anti-Blackness and white supremacy, systemic racism may include the school-to-prison pipeline, institutional racism can take the form of police brutality, and overt racist actions exemplify interpersonal racism. While reparations have been granted to Black Americans under specific circumstances where culpability and harm are undeniable (e.g., the Tuskegee Syphilis Study when Black men with syphilis were knowingly medically neglected), the exploitation and marginalization of the greater Black community for more indirect forms of structural and systemic racism remain largely unaddressed [11].

Despite the well-established history of the harms caused by slavery and Jim Crow, attempts to address their long-lasting impacts have often been uneven and disorganized [6, 8]. The evolution from focusing on historical injustices to contemporary reparations initiatives marks a significant shift in understanding racial harm, not just as a legacy of the past, but as an ongoing contributor to inequality. Today’s reparations efforts aim to tackle the lasting effects of historical exploitation, particularly concerning wealth and health disparities that have intensified over many years. This shift indicates a movement away from merely acknowledging these issues symbolically to implementing structured and policy-oriented responses to systemic racism. Moreover, it remains unclear how well the current reparations initiatives proposed by stakeholders to address these forms of racism are coordinated [12]. This lack of clarity creates further challenges in discerning what types of reparations should be demanded and how they ought to be administered.

### Public Health Critical Race Praxis

Public Health Critical Race Praxis (PHCRP) offers a robust framework for examining and addressing socioeconomic health inequities stemming from systemic racism [12]. PHCRP merges Critical Race Theory with public health initiatives. This theory highlights racism as a fundamental cause of health disparities and advocates for systemic changes to eradicate racial inequality. Additionally, PHCRP bridges the gap between research and practical application by presenting a comprehensive set of guiding principles that inform the design and evaluation of interventions, such as those focused on reparations.

One of the core premises of PHCRP is that racism is permanent [12]. Additionally, PHCRP advocates for the explicit recognition and investigation of racism in public health research, policies, and practices, firmly opposing colorblind approaches that overlook the role of race in shaping outcomes. Furthermore, PHCRP advocates call for a critical examination of historical events such as slavery and segregation, along with their ongoing impacts on the health and socioeconomic realities of Black Americans.

Proponents of PHCRP seek to amplify the voices and lived experiences of those most harmed by systemic racism, which must be front and center [12]. In doing so, it abandons traditional top-down approaches for participatory, community-based methods that cater to the needs, perspectives, and competencies of Black communities. Furthermore, this is an intersectional framework that considers the overlapping nature of sexism, classism, and homophobia that impact Black women, poorer Black Americans, and LGBTQ + Black Americans. Consequently, any reparative measures must confront these intersecting systems of disadvantage for tangible equity to be accomplished.

Lastly, proponents of PHCRP profess to be committed to action and transformation and do not believe in piecemeal or merely symbolic solutions but in transformational systemic change [12]. Using PHCRP, they critically examine public health interventions that address only symptoms without addressing their causes, instead advocating for large-scale structural reforms that redistribute power and resources to promote the “deracialization” of society. Guided by these premises, PHCRP offers an uncompromisingly strict and action-oriented framework through which health disparities, justice, and equity can be promoted across all levels of society.

### Socioeconomic and Health Inequality

PHCRP, along with cumulative disadvantage theory, helps explain the white wealth generated from slavery and the socioeconomic disparities in income, wealth, and education that Black Americans have inherited [13–16]. These theories illustrate the cascading and worsening nature of inequality over generations as mutually reinforcing mechanisms—such as residential segregation, underfunded schools, over-policing, and lack of accessible healthcare—trap Black American families in cycles of disadvantage [17–20]. Many of these mechanisms emerged after slavery and contributed to the growing socioeconomic gap between Black Americans and white Americans. As of 2023, white Americans possess 5 to 10 times the wealth of Black Americans, with 35–45% of this wealth being inherited. Reparations advocates argue that the absence of measures following the abolition of slavery has allowed socioeconomic inequality to persist and worsen due to subsequent forms of racism [1].

Mechanisms such as residential segregation and access to quality education contribute to socioeconomic inequality and are social determinants of health inequality [21–24]. Black Americans are significantly more likely to develop heart disease, hypertension, cancer, and comorbidities. While these comorbidities are linked to more recent forms of racism in the US timeline, there is also a direct link between descendants of slavery and slavery as an institution [25–28]. Reece found that the current life expectancy of Black Americans today is significantly lower for those living in southern counties in the U.S., where slavery was widely practiced, even when controlling for factors such as income and healthcare [28]. Like socioeconomic inequality, health inequality accumulates across the life course and over generations, contributing to Black Americans dying four years sooner than their white counterparts [21–24]. The persistence of upstream structural and systemic racism, as well as more downstream forms of racism like institutional and individual discrimination, suggests that reparations following slavery were a missed opportunity to prevent socioeconomic and health inequality seen today [16]. These socioeconomic and health disparities illustrate PHCRP’s assertion that systemic racism operates as a fundamental cause of inequity, shaping generations of disadvantage [12].

With recent reparative discourse, there has been a growing body of literature regarding the potential ability of reparations to mitigate health and socioeconomic inequality among Black Americans. Richardson et al. estimate that reparations, in the form of direct payments, could potentially reduce the reproduction of the COVID-19 virus by 31–68% [5]. Similarly, Himmelstein et al. estimate that the four-year gap in life expectancy between Black and white Americans could be rectified by administering over $800,000 per Black American household [29]. This, of course, would not only benefit the health of Black Americans but also bring the question of the overall public health benefits of reparations to light. CRT emphasizes the importance of these measures in redistributing resources and addressing cumulative disadvantage while critiquing the fragmented nature of many current reparations efforts.

Outside of the COVID-19 pandemic, Bassett and Galea suggest three pathways by which reparations could theoretically close the gap in health disparities between Black and white Americans: (a) increasing and expanding socioeconomic resources such as safe neighborhoods, better schools, and clean air); (b) reducing the physical and psychological stress that Black Americans are subjected to; and (c) reducing the cumulative disadvantage in health and wealth experienced over generations [30]. Outside of Richardson et al.’s work, however, little research exists that quantifies the potential impact of reparations on the health of Black Americans [5].

### Organizational Initiatives

With the understanding that reparations can theoretically and practically limit socioeconomic and health disparities among Black Americans, many modern organizations have established reparative initiatives, demanding the US government take action to rectify generations of socioeconomic and health inequality [4, 31, 32]. For example, the National African American Reparations Commission (NAARC) created a 10-point Reparations Plan in 2015 [31]. The plan includes measures beyond direct payments, such as local, state, and federal governments administering cash payments to qualifying Black Americans. The NAARC [33] also includes reparative strategies to modify educational, housing, and healthcare infrastructure in the USA. Other reparative initiatives include the National Association for the Advancement of Colored People’s (NAACP) reparations plan and the Movement for Black Lives (M4BL) Reparations Now TOOLKIT [31, 34].

The plans proposed by the NAARC, NAACP, and various other groups have been largely ineffective, failing to secure either partisan or bipartisan support from local, state, and federal lawmakers [32, 34]. With only 18% of white constituents supporting reparations, even more progressive white policymakers have shown little interest in considering these proposals in their current form [34]. Opposition to reparations often stems from concerns about fairness and feasibility [33, 35]. Critics argue that it is unjust to hold present individuals accountable for past wrongs committed by earlier generations, particularly when many contemporary Americans lack direct ties to slavery. Furthermore, there are logistical issues related to determining eligibility for reparations and identifying potential funding sources, with some concerned that these initiatives could exacerbate social divides or impose undue financial burdens on taxpayers. Additionally, some skeptics question whether reparations would genuinely address systemic inequities or merely serve as symbolic gestures that fail to tackle the underlying issues. However, as legislative bodies become more racially and ethnically diverse, the pressure from organizations and activists on primarily Black American legislators to advocate for reparations has intensified [36].

### Contemporary Reparative Legislation

This pressure has also increased with the rise of the Black Lives Matter Movement [3, 37, 38]. The police murders of Michael Brown, Philando Castille, Tamir Rice, Eric Garner, and countless other Black Americans reignited the fight for large-scale, cumulative reparative measures in 2014. The call for reparations has been further inflamed by the murders of Breonna Taylor and George Floyd years later during the height of the pandemic. Activists such as Ta’Nehisi Coates, William Darity, and Randall Robinson helped galvanize the issue into the national discourse, not only making a case for reparations for systemic violence such as police brutality but outlining the debt that is owed to Black Americans, in general, regardless of whether they were victims of individual acts of overt racial violence [1, 3, 39].

This discourse inspired Evanston, Illinois, a suburb of Chicago, to establish a $10 million program to provide $25,000 to eligible Black Americans over ten years [40–42]. Taxes on newly legalized marijuana sales mainly fund the reparations program in Evanston. To qualify, Black Americans must have resided in Evanston prior to 1969, when redlining was at its peak. As of March 2025, Evanston has distributed $5 million of the promised $10 million [42]. However, the city is now facing a lawsuit from a conservative legal group claiming racial discrimination against non-Black residents.

Nevertheless, Evanston’s initiatives have had a significant impact on the wider Chicago area [43]. Following the police murder of George Floyd in 2020, Chicago’s City Council established a Subcommittee on Reparations to determine how the city should provide reparations to ADOS. The committee’s initial efforts were largely unproductive. Recently, though, the city’s mayor has pledged $500,000 in 2024 as seed money to revive the committee’s efforts regarding reparations for Chicago’s residents [44]. As of May 2025, a task force has been created, comprised of 25 members appointed by the Mayor’s Office and the City Council’s Black Caucus, with the remaining 15 positions filled through a public application process.

Other recent legislative efforts to bring reparations to fruition include California’s Task Force to Study and Develop Reparation Proposals for Black Americans, which was formed in 2020, as well as former US Congresswoman Cori Bush’s Reparations Now Resolution introduced in 2023 [45, 46]. After several years of public hearings, California’s Reparations Task Force has voted to recommend that the state apologize for its role in upholding slavery as well as direct payments to Californians whose ancestors were enslaved. The amount owed would vary greatly depending on the time lived in the state; however, only ADOS are eligible. Considering that 80% of Black Californians are ADOS, it is estimated that 2.8 million Black Americans could receive a payment if passed by the state legislature [47].

Similar to California’s task force, former Congresswoman Bush’s Reparations Now Resolution seeks restitution for slavery at the federal level [48]. The Reparations Now Resolution supports other federal reparative initiatives, such as H.R. 40, and has been endorsed by 300 organizations [49]. Whether or not California’s or Congresswoman Bush’s efforts will be successful, both in the fight for reparations and whether they will adequately address socioeconomic and health inequality among Black Americans, remains to be seen. Even so, the initiative has sparked a chain reaction at the state and municipal levels, with stakeholders rapidly crafting initiatives over the last year and continuing to do so in real time [50, 51].

### Purpose

As calls for reparations intensify and persist, it is crucial to identify the key elements embedded in the reparative plans and bills proposed by stakeholders [42, 46]. This is essential to stimulating ongoing dialogue and collaboration among stakeholders and the broader public to navigate the complexities of reparations, foster unity, and ultimately mitigate socioeconomic and health inequality among Black Americans.

Recent reparative initiatives, like Congresswoman Cori Bush’s H.R. 40 bill and Evanston’s local reparations plan, highlight the lack of a harmonized approach [42, 46]. For example, the federal initiative supports “reparations in all necessary forms” and is not limited to ADOS, whereas Evanston’s plan focuses on direct payments to descendants of American slavery. This disjointedness creates a gap that the proposed research seeks to address [43]. Without a unified approach, these efforts risk operating in silos rather than contributing to a more comprehensive reparative agenda.

To gain a comprehensive understanding, stakeholders and the general populace need transparency on the specific issues reparations are designed to address, proposed methods of restitution, and the overarching vision that guides these initiatives. Additionally, the reparations proposals put forth should be evaluated in terms of their ability to address socioeconomic and health inequality. Therefore, the purpose of this rapid review is to determine (a) the eligibility criteria and grounds for reparations, (b) the extent to which recommendations to address socioeconomic and health inequality align with PHCRP, and (c) the ultimate goals of each plan considering their historical and contemporary context [12].

## Method

### Search Strategy

This is a mixed-method rapid review that utilized Dobbin’s Rapid Review Guidebook to employ qualitative and limited quantitative comparative analyses of reparative initiatives [52]. The rapid review took place in September of 2024 and was updated in December 2024 and June 2025 in response to revisions requested by journal reviewers. A rapid review, as opposed to a systematic review, was chosen due to the rapidly evolving and fluctuating nature of the discourse, legislation surrounding reparations for Black Americans, and the impending 2024 presidential election. This means that the landscape of reparations initiatives is marked by considerable dynamism, with new initiatives consistently emerging and existing ones undergoing rapid changes or experiencing periods of stagnation. Due to time constraints and the inherently fluid nature of these initiatives, as well as the impending presidential election, a comprehensive systematic review was deemed impractical. The review began by explicitly outlining the following primary research questions:

1. What are the eligibility criteria and overall grounds for reparations?
2. How do the goals of reparative initiatives differ by level of government, if at all?
3. What types of reparations are stakeholders recommending, and to what extent can they address socioeconomic and health inequality under PHCRP?

Secondary questions included:

1. What is the political ideology underlining reparative initiatives?
2. How coordinated are the modern reparations plans and bills proposed by stakeholders, and what challenges do they face?

Next, the investigator conducted an extensive online search using keywords and phrases related to reparations and racial justice. Boolean operators (AND, OR) were used on Google, Google Scholar, and academic databases using EBSCOhost to refine search queries (Table 1). For example, (“reparations plan” OR “recommendations,” OR “redress”) AND (“Black Americans” OR “African Americans” OR “American Descendants of Slavery”) terms were used.

**Table 1:** Sources, search terms, and EBSCOhost filters were applied in the rapid review

Source	Search term	EBSCOhost filters
EBSCOhost	(“Reparations” OR “Reparative initiatives” OR “Reparations Plan” or “Reparations Initiative” OR “Reparations Plans”) AND (“Black Americans” OR African Americans OR American Descendants of Slavery)	• Excluded due to not being in English (6) • Excluded due to being published before 2000 (7)
Congress.gov	(“Reparations”) AND (“Black Americans” OR “African Americans”)	• Excluded congressional records (586) • Excluded committee meetings (107)
State and Local Search (Google and State Websites)	(“STATE* reparations” OR “reparations initiatives” OR “reparations task force” OR “STATE* reparations plan” OR “local government reparations”) AND (“Black Americans OR African Americans OR American Descendants of Slavery”)	Gray literature
Organizations	(“Reparations organizations,” OR “Black advocacy organizations”) AND (“Black Americans OR African Americans OR American Descendants of Slavery”)	Gray literature

Legal databases such as Congress.gov and state legislative databases were searched for bills, resolutions, legislative initiatives, and other grey literature containing concrete demands and reparations plans [53]. Keywords and phrases needed modification based on the search engine used. For instance, Congress.gov cannot accommodate multiple Boolean operators. Snowball sampling was also utilized, particularly in the grey literature that often inspired and cited other reparative initiatives.

### Inclusion and Exclusion Criteria

**Fig. 1:**
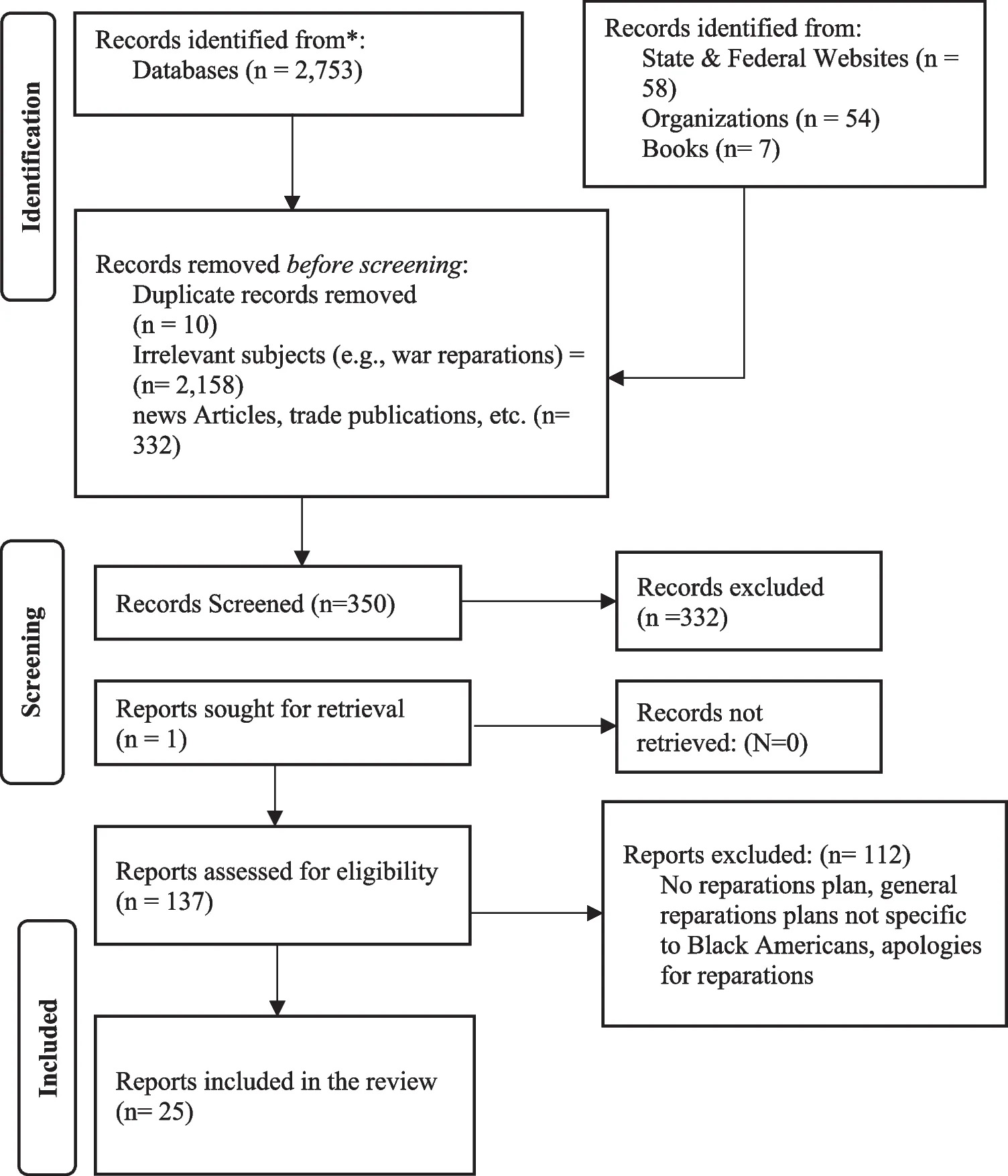
Identification of studies via databases and other methods

The initial search yielded 2,753 documents to be screened (Fig. 1) [54]. Before screening database articles, search engine features such as removing duplicates, utilizing EBSCOhost to filter out irrelevant types of publications (e.g., newspaper articles), and excluding irrelevant topic areas were employed. After this, the following inclusion and exclusion criteria were applied to initiatives, following the screening of titles and abstracts. Initiatives put forth by local, state, and federal legislators, organizations, and less formulated groups were included. To be included in the analysis, the initiative had to have a list of demands or recommendations, an argument about the types of reparations that should be implemented, and/or a step-by-step reparations plan. Pieces that solely argued *for* reparations but did not include a list of demands, recommendations, or a reparations plan were excluded because the purpose of this analysis is to outline what stakeholders are demanding, not whether reparations should be implemented.

This analysis surrounds reparations for indirect forms of racism, such as the accumulated wealth and health inequality from being a descendant of slavery, for which culpability is obscured. Therefore, lawsuits that demanded compensation for direct acts of racial violence and discrimination were excluded. The rationale for this exclusion is that lawsuits typically involve legal actions filed in courts to seek compensation or justice for specific individuals or groups. They are often distinct from bills, resolutions, or plans proposed by legislators or organizations, which involve proposed laws and policies.

Other exclusions include apologies made by state, local, and federal legislators and institutions that were not accompanied by some other form of restitution. For example, Virginia, Maryland, and the University of Alabama have apologized without offering any other form of reparations. [55–57]. Apologies acknowledge historical injustices, express regret, or convey empathy but do not involve concrete policy measures, which is the primary focus of this reparations analysis. Lastly, reparations paid by some institutions and organizations, such as JP Morgan Chase and Georgetown University, were excluded as the reparations were paid without a plan put forth by Black Americans [58, 59]. Additionally, the included initiatives primarily focused on Black and African Americans and were assembled during or after the year 2000. The year 2000 was selected as a cutoff point as contemporary reparations plans are more likely to reflect current societal debates, political climates, and public opinions.

### Qualitative Data Extraction and Analysis

#### Units of Analysis

A total of 25 initiatives were identified for qualitative analysis. After the initiatives were consolidated, the data analysis and familiarization process began. Many of the demands and plans for reparations followed an argument for why reparations should be implemented in their respective state or municipality. These demands and plans were also commonly part of resolutions that called for the state or municipality to form a task force to study or take some action on reparations.

To address the main aims of this study, the units of analysis were determined by the parameters of the demands of the plan. If the plan was part of a larger initiative, only the plan itself was examined. For example, California’s reparations plan is included in a much larger report investigating the state’s role in upholding slavery and the debt it owes to Black Californians today [45]. Therefore, the broader initiative was used to contextualize the reparations plan but was not necessarily included in the analysis.

#### Qualitative Coding

These units of analysis were transferred into NVivo, a qualitative data management software. Coding started with open coding, where the smaller units of data were given descriptive labels that captured such key concepts as eligibility criteria, reparative measures, and systemic barriers. A broad set of codes generated in this initial phase then needed refining during selective coding to identify the core categories and the nature of the relationship between them. This iterative process eventually led to overarching themes surrounding eligibility; goals and grounds for reparations; types of reparations; and coordination challenges that were identified.

To maintain methodological rigor, the included initiatives were evaluated primarily for content relevance instead of traditional study quality, considering the nature of the sources (e.g., legislative resolutions, advocacy plans, municipal reports). These are not empirical studies but publicly available documents presenting policy demands, which makes traditional bias assessment tools (e.g., CASP or AMSTAR) less applicable. However, during the coding process, efforts to assess ideological positioning, stakeholder representation, and transparency of demands served as a proxy for bias assessment. Specifically, the use of PHCRP as a critical framework helped identify whether the initiative acknowledged structural racism, centered marginalized voices, and critiqued colorblind policy framings.

##### Evaluation via PHCRP

PHCRP was used as an evaluative framework during the coding process to examine how well reparations initiatives address socioeconomic and health inequality [12]. Several of the foundational elements of PHCRP were considered:

1. **Racism as a Fundamental Cause:** The initiatives were evaluated for their acknowledgment of racism as a primary cause of health and socioeconomic inequities.
2. **Historical and Contemporary Context:** The review examined the extent to which reparations are framed within the histories of slavery and systemic racism, as well as their ongoing impacts on contemporary society.
3. **Centering Marginalized Voices:** The degree to which efforts centered the voices and experiences of Black Americans, especially those who have been most harmed by systemic racism.
4. **Intersectionality:** How well initiatives considered the layering oppressions such as misogyny, ableism, and ageism that impact subpopulations of Black Americans.
5. **Critique of Colorblindness:** Initiatives were evaluated regarding their explicit focus on racial inequity, intentionally avoiding framings that obscure the role of race.
6. **Commitment to Structural Change:** The review assessed how an initiative fosters transformative, system-implicating changes that dismantle inequitable systems, such as capitalism and structural racism, which are mutually reinforcing.
7. **Redistribution of Power and Resources:** The review assessed whether reparations efforts are designed to redistribute power and resources, addressing disadvantages accumulated over time and achieving equity.

Because the coding was conducted by a single researcher, inter-coder reliability testing was not performed. To support the validity of the findings in the absence of multiple coders, reflexive memoing was used throughout the process to document coding decisions and analytical rationale. Memoing during the coding process facilitated the recording of reflections, tracking of decisions, and reducing any potential biases.

### Comparative Extraction and Analysis

Given the inherently conceptual nature of numerous reparations initiatives and the scarcity of quantitative data (such as per capita funding or population coverage), this study employed a limited comparative methodology to systematically analyze qualitative data. To prepare for comparative analysis, the various characteristics of the initiatives were quantitatively coded in SPSS, a quantitative data management software, after the open coding phase as qualitative themes began to emerge.

To address the first primary research question, eligibility requirements were categorized into six groups: Diasporic (i.e., Black Americans and more recent immigrants in a particular locale or state); ADOS; ADOS and Foundational Black American Residents (i.e., those living in a specific state or local area during a particular time period) and Descendants of Residents; To Be Determined; and Black Americans and other Marginalized Groups (i.e., Black Americans living in a specific state/local along with another group such as Indigenous Americans). Types of reparations were also coded and organized into eight broad categories to address the second and third research questions: Direct Payments; Truth Seeking and Acknowledgment; Restitution and Protection of Land/Property; Health Services; Educational and Vocational Training; Criminal Justice Reform; Reparative Infrastructure; and Black Business Protection and Economic Development.

In addition to the eligibility requirements and types of reparations included in the plans, initiatives were classified as international, federal, state, local, or organizational, depending on the organizing body (e.g., the US Congress, a nonprofit organization) that put forth the initiative. Moreover, the goals and grounds were consolidated into seven categories, including reparations for slavery and Jim Crow, to address socioeconomic and health inequalities, and to support other reparative initiatives. Lastly, based on the qualitative results discussed below, the overall ideology was coded as socially liberal or progressive. Social liberalism focuses on balancing individual liberty with social justice through moderate reforms within the existing system, while progressivism advocates for more extensive reforms to address deep-seated social and economic inequalities [59]. These distinctions were applied by the researcher when interpreting each initiative’s ideological stance, based on the initiative’s stated goals, language, and proposed methods of reparative justice. The final characteristics were then reviewed for consistency and alignment with the study’s objectives.

The dataset consisted of the 25 initiatives identified in the comparative analysis, encompassing various governance levels (e.g., international and local). The six categories of eligibility requirements were coded categorically in SPSS as follows: Diasporic = 1, ADOS only = 2, ADOS and Foundational Black American residents and/or their descendants = 3, and so on. The eight types of reparations (e.g., Direct Payments, Truth Seeking and Acknowledgment) were binarily coded as either 1 = present or 0 = absent. Other coded variables include the goals and grounds (e.g., slavery = 1, Jim Crow = 2, redlining = 3etc.), ideology (e.g., socially liberal = 1, progressive = 2), and barriers (e.g., funding = 1, lack of political support = 2, constituent backlash = 3). This coding method effectively converts qualitative themes into a dataset suitable for comparative analysis.

## Results

### Results Overview

Many of the local and state initiatives included in the analysis were formed in allegiance with former Congresswoman Bush’s H.R. 40 and H.R. 414 resolutions to bring reparations to fruition at the federal level (Table 2; see Appendix 1 additional details) [46, 60]. Multiple states and local initiatives are in the process of drafting plans but do not yet have a reparations plan in place, and therefore, were excluded from the analysis. However, many reparations task forces and initiatives are being formed in real time. Wilmington, Delaware; Philadelphia, Pennsylvania; the State of Connecticut; and numerous institutions, such as universities and churches, have assembled and passed resolutions to form task forces to address reparations this past year alone [50, 51]. This includes efforts to collaborate with research institutions to study the history of racial oppression in the respective state or municipality. The work they have done so far, such as Boston, Massachusetts, lawmakers putting out calls for research partners to study the lasting impact of slavery in the city, provides a pathway for creating a concrete reparations plan in the future [60].

**Table 2:** Summarized information on reparations initiatives

Organizing body	Year	Name	Location
International			
United Nations General Assembly [61]	2016	Implementation of the International Decade for People of African Descent	International and decentralized effort
Federal			
118th Congress [62]	2023	H.R. 414 Reparations Now Initiative	Washington, D.C
119th Congress [63]	2025	H.R. 414 Reparations Now Initiative	Washington, D.C
State			
African Descent-Citizens Reparations Commission [64]	2022	20 ILCS 405/405–540	Illinois, U.S
California Reparations Task Force [45]	2023	California Reparations Report	California, U.S
Oregon General Assembly [65]	2021	Senate Bill 619	Oregon, U.S
Local			
African Heritage Reparations Assembly [66]	2023	Final Report	Amherst, MA
Asheville Community Reparations Commission [67]	2020	Community Reparations Commission Draft Recommendations	Asheville, NC
Athens Reparations Action [68]	2021	The Linnentown Resolution	Athens, GA
Black Austin Coalition [69]	2021	Resolution No. 20210304–067	Austin, TX
Equity Empowerment Commission [43]	2019	Recommendations on actions to address wealth and opportunity gaps	Evanston, IL
Providence Municipal Reparations Commission [70]	2022	Report of the Providence Municipal Reparations Commission	Providence, RI
San Francisco African American Reparations Committee [71]	2023	San Francisco Reparations Plan 2023	San Francisco, CA
Shelby County Commission Reparations Committee [72]	2023	Shelby County Reparations Resolution	Shelby County, TN
South Bend Common Council Reparatory Justice Commission [73]	2023	Bill No. 22–61 A Resolution Calling for Reparatory Justice	South Bend, ID
St. Paul Reparations Legislative Advisory Committee [74]	2022	Resolution 21–77	St. Paul, MN
Organizational			
American Descendants of Slavery Advocacy Foundation	2016	ADOS Reparations Agenda	Decatur, Georgia
Brookings Institute 2020 [75]	2020	Why We Need Reparations for Black Americans	Washington, DC
Coming to the Table (CTTT) [76]	2021	A Guide to the Reparations Movement	Oakland, California
Decolonizing Wealth Project (DWP)	2018	Seven Steps to Healing	New York, New York
The Civil Rights and Restorative Justice Project [77]	2023	A Toolkit for Policymakers and Advocates	Boston, MA
The Episcopal Diocese of Maryland	2019	Diocese Reparations Program	Baltimore, MD
The Movement for Black Lives	2016	M4BL Reparations Now Toolkit	Decentralized Coalition
National African American Reparations Commission	2015	10-Point Reparations Program	Decentralized Coalition
Vermont Racial Justice Alliance [78]	2021	Burlington’s Racial Equity Strategic Roadmap	Burlington, VT

Appendix 1 provides additional details regarding eligibility requirements, current status, etc. Each initiative is listed in the references section

### Theme 1: ADOS, at Minimum, Should be Eligible for Reparations

The first research question surrounded the eligibility of reparations. Fifteen out of the twenty-five reparations initiatives largely surrounded, at minimum, reparations for ADOS (Table 3). Seven initiatives reviewed were exclusive to ADOS, including the only federal initiative in the quest for reparations. For example, the African Descent-Citizens Reparations Commission’s statute and the American Descendants of Slavery Advocacy Foundation’s reparations report were crafted in response to hundreds of years of chattel slavery in the USA [64, 79].

**Table 3:** Frequencies regarding eligibility

Eligibility	Frequency	Percent
Diasporic, including ADOS	4	17%
ADOS only	7	25%
ADOS, Foundational Black American residents, and/or their descendants	3	13%
Foundational Black American residents and/or their descendants only	4	17%
To be determined	5	21%
ADOS and other marginalized groups	2	8%

Diasporic refers to people of African descent living in the USA who may or may not consider themselves as Black Americans or ADOS. For example, people who are from the Caribbean or Africa or whose ancestors migrated from the Caribbean or Africa, not the Transatlantic Slave Trade. Foundational Black Americans refer to people who may or may not be ADOS but who have lived in a particular state or locale for several generations. For example, California requires potentially eligible participants to be ADOS or to be descended from those who lived in the state prior to 1900. Moreover, some locales, such as Athens, GA, require applicants to be foundational (i.e., lived in the locale before or during a designated time) to the city or a descendent of someone who was but is not necessarily limited to ADOS. Lastly, two initiatives included reparations for ADOS and other marginalized groups such as Indigenous Americans

The bulk of initiatives included more recent Black immigrants, Foundational Black Americans, and Indigenous Americans, in addition to ADOS, who have been subjected to racialized and ethnic violence [50]. The common logic here was that while ADOS should receive reparations for the atrocities of slavery, other Black groups were subjected to similar atrocities in different parts of the globe. For example, the colonization of Africa or slavery in the Caribbean. Moreover, those who are immigrants or who are descendants of Black immigrants face much of the same anti-Blackness as ADOS do, while other non-Black groups have also faced the brunt of white supremacy. For example, the Decolonizing Wealth Project’s mission is to redistribute wealth from white Americans taken from Indigenous Americans and Black Americans in the form of land theft and unpaid labor [80].

**Fig. 2:**
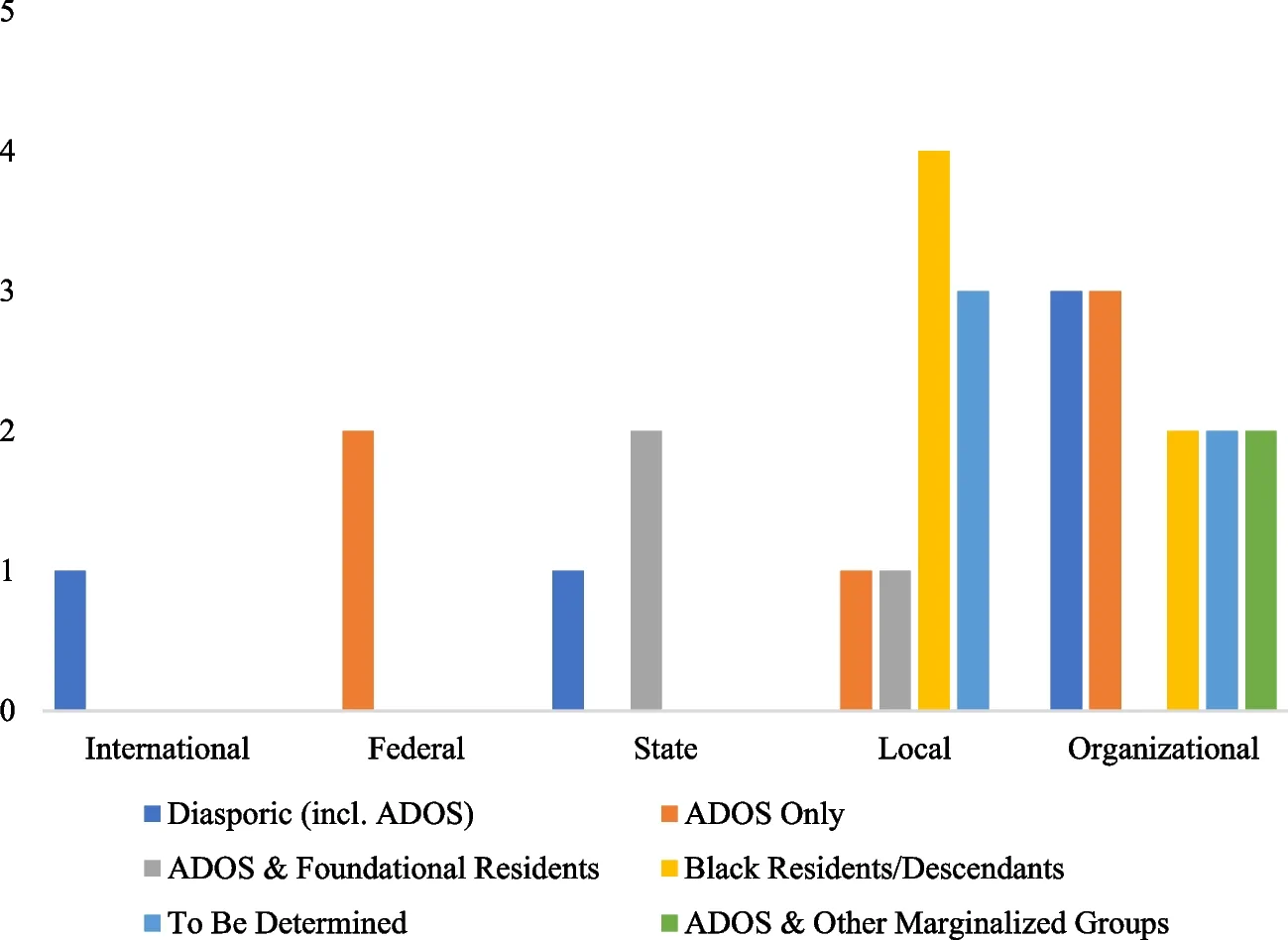
Eligibility requirements by level of government

Regardless of whether other groups were included, local and state initiatives tended to be more specific with their eligibility requirements than federal, international, or organizational initiatives (Fig. 2). Local and state initiatives, intuitively, often required that Black Americans, both ADOS and non-ADOS and their descendants who live in the respective municipality or state to, be eligible for reparations. However, municipalities such as Evanston, Illinois, and the state of California have time restrictions, wherein citizens or their ancestors must have lived in the region [42, 45]. For example, Evanston residents must have lived in the city from 1919 to 1969 to be eligible, while the California Reparations Taskforce considers non-ADOS eligible so long as they are descendants of Californians who lived in the state before 1900.

The distinction in eligibility is the grounds or reasoning for reparations. Evanston is interested in administering reparations to citizens who were most impacted by redlining during its height in the city [41]. Conversely, California is interested in administering reparations to citizens who were foundational to the establishment of the state, particularly as they fled from slavery and racial violence, such as lynchings in other parts of the country [45]. Organizational initiatives were less concerned with citizenship or residency because, even though they may be headquartered in a specific part of the country or are decentralized, their plans often focus on reparative liberation for Black individuals across the country [31].

Overall, a distinction exists between governmental initiatives at the international, federal, state, and local levels and those of organizations. For example, governmental initiatives seem to correspond with the primary transgressions or “original sins” against Black Americans in their respective country, state, or city. In the case of the United States government, this refers to the establishment, facilitation, and extensive profit from slavery. For California and Illinois, it relates to the indirect benefits gained from slavery and racial discrimination. Local initiatives address issues such as urban renewal, redlining, and other forms of systemic, institutional, and interpersonal racism that have occurred or continue to occur in those specific areas [42, 45, 60].

Regarding organizational initiatives outside of local, state, or federal governments, the basis for reparations was much broader and less specific. Stakeholders urged policymakers at all levels, as well as institutions in healthcare, education, and the private sector, including corporations, to coordinate and consider their recommendations and demands for reparations. For instance, the M4BL Toolkit and NAARC’s 10-point plan recognize the centuries of widespread anti-Blackness, violence, and racism in various ongoing forms as a combined responsibility of both public and private sectors in supporting reparations [31, 32].

### Theme 2: The Overall Goals and Grounds of Reparations Differ Between Governmental and Organizational Initiatives

The second research question of this review was to determine why reparations initiatives were created and what stakeholders sought to accomplish (Table 4). Similar to eligibility, this depended on whether the plan originated within a governmental body such as a state or local task force or a separate organization. At the international level, the U.N. General Assembly initiative tackles all major categories, including slavery, Jim Crow laws, and recognition, highlighting a global understanding of systemic injustices [61]. In contrast, federal initiatives, such as those from the US 118th Congress, tend to zero in on issues related to slavery and Jim Crow, mainly focusing on acknowledgment while somewhat neglecting pressing contemporary challenges like socioeconomic inequality and mass incarceration [46].

**Table 4:** Goals and grounds of initiatives by level of government

Overseeing body	Slavery	Jim crow	Redlining, urban renewal, and housing	Socioeconomic, educational, and health inequality	Criminal legal issues	Acknowledgment and preservation	Support other initiatives	
International								
United Nations General Assembly	X	X	X	X	X	X		
Federal initiatives								
US 118th Congress	X	X				X		X
US 119th Congress	X	X				X		X
State initiatives								
African Descent- Reparations Commission	X		X	X				
California Reparations Task Force	X	X	X	X	X	X		X
Oregon State Legislature	X							
Local initiatives								
African Heritage Reparations Assembly				X	X	X		
Asheville Community Reparations Commission	X	X	X	X	X			
Athens Reparations Action			X			X		X
Equity Empowerment Commission		X	X	X				
Providence Reparations Commission	X	X	X	X		X		
San Francisco Reparations Committee		X	X	X	X	X		
Shelby County Reparations Committee			X	X	X			
South Bend Justice Commission			X	X		X		
St. Paul Reparations Committee	X	X	X	X		X		X
Organizational initiatives								
ADOS Foundation	X		X	X		X		
Brookings Institution			X	X				
CTTT	X	X	X	X	X	X		
DWP	X	X	X	X	X	X		
The Civil Rights Justice Project	X	X	X	X		X		
The Episcopal Diocese of Maryland				X	X	X		
The M4BL	X	X	X	X	X	X		X
The NAARC	X	X	X	X	X	X		
Vermont Racial Justice Alliance				X	X			

The names of the initiatives have been consolidated for readability

At the state level, initiatives like the California Reparations Task Force demonstrate a broader range of aspirations, addressing systemic inequalities and concerns related to housing and policing, as well as state-specific issues [45]. Local initiatives reflect even more variation, as seen with the Asheville Community Reparations Commission, which takes a broader approach compared to the Athens Reparations Action, which is concentrated on redlining and urban renewal [67, 68]. This diversity underscores the need to tailor reparative actions to local histories and contexts.

Regarding organizational initiatives, they often present highly comprehensive strategies to tackle systemic issues. M4BL and the NAARC encompass a wide array of topics, from slavery and Jim Crow to systemic inequalities, housing, and mass incarceration, all while promoting acknowledgment and fostering other supportive initiatives [4, 32]. Throughout these efforts, there is a robust emphasis on acknowledgment and preservation, reflecting a shared recognition of historical injustices as a crucial element of reparative actions. Nevertheless, some critical gaps exist. For instance, while mass incarceration is mentioned in many initiatives, it does not receive consistent priority at the federal and international levels. Additionally, state and federal stakeholders often fail to explicitly back interconnected initiatives, unlike organizations such as NAARC and M4BL, which actively push for collaborative frameworks.

From a PHCRP standpoint, the differences between government and organizational initiatives highlight both potential common ground and significant gaps [12]. Some local initiatives effectively align with PHCRP’s approach, emphasizing the importance of understanding reparations within the context of historical and ongoing systemic inequalities. However, the two federal and international efforts do not meet this standard. The federal initiative, for example, overlooks crucial aspects of PHCRP, such as entirely rejecting colorblindness and directly addressing how race contributes to ongoing disparities in socioeconomic and health outcomes.

On the other hand, many organizational initiatives do a better job of aligning with PHCRP’s core principles, focusing on redistributing power and resources while confronting systemic racism [12]. Even so, these organizational efforts could still enhance their impact by building coalitions and intentionally supporting other initiatives.

#### Subtheme: Social Liberalism vs. Progressivism

In addition to differences in goals and grounds for reparations, organizational initiatives tended to adopt a more radical approach, employing stronger language that emphasizes revolution, anti-racism, and dismantling systems of oppression. These initiatives are ultimately aimed at Black liberation, reflecting a *progressive* stance compared to the *socially liberal* reparative frameworks often seen in governmental initiatives [59]. For instance, the African Heritage Reparations Assembly proposed funding an endowment through marijuana sales, while the NAARC advocated redistributing land to a National Reparations Trust Authority [32, 66]. Governmental initiatives, while largely socially liberal, exhibit progressive elements, such as H.R. 414’s proposal to establish the United States Commission on Truth, Racial Healing, and Transformation, which aims to examine the intersection of racism and policy [46]. However, most governmental efforts remain focused on incremental reforms and avoid addressing the broader systemic inequities perpetuated by corporations, institutions, and present-day white Americans.

Applying the principles of PHCRP reveals essential distinctions between these approaches [12]. Organizational initiatives more explicitly tackle systemic racism, advocating for comprehensive measures such as wealth redistribution, land allocations, and direct accountability from white Americans and institutions for slavery and its enduring effects. This aligns with PHCRP’s core tenet of rejecting colorblindness and confronting the ongoing impact of race on power, resources, and health disparities. By contrast, governmental initiatives often avoid implicating present-day structures and actors, a cautious approach that diverges from PHCRP’s emphasis on addressing contemporary inequities head-on.

While both initiatives incorporate PHCRP’s attention to historical and contemporary contexts, organizational approaches are more closely aligned with its goal of redistributing power and resources and tackling systemic racism as a root cause [12]. To further strengthen alignment with PHCRP, stakeholders across initiatives could adopt a more radical perspective that actively challenges exploitative economic systems, such as capitalism, rather than merely reallocating existing resources. Moreover, integrating intersectionality more explicitly into these initiatives would ensure that reparative efforts address the intersecting oppressions faced by marginalized groups within Black communities, such as Black women, LGBTQ + individuals, and people with disabilities. This would create a more comprehensive and egalitarian framework that is fully consistent with PHCRP’s vision for dismantling systemic inequities.

### Theme 3: Reparations Extend Far Beyond Direct Payments

**Fig. 3:**
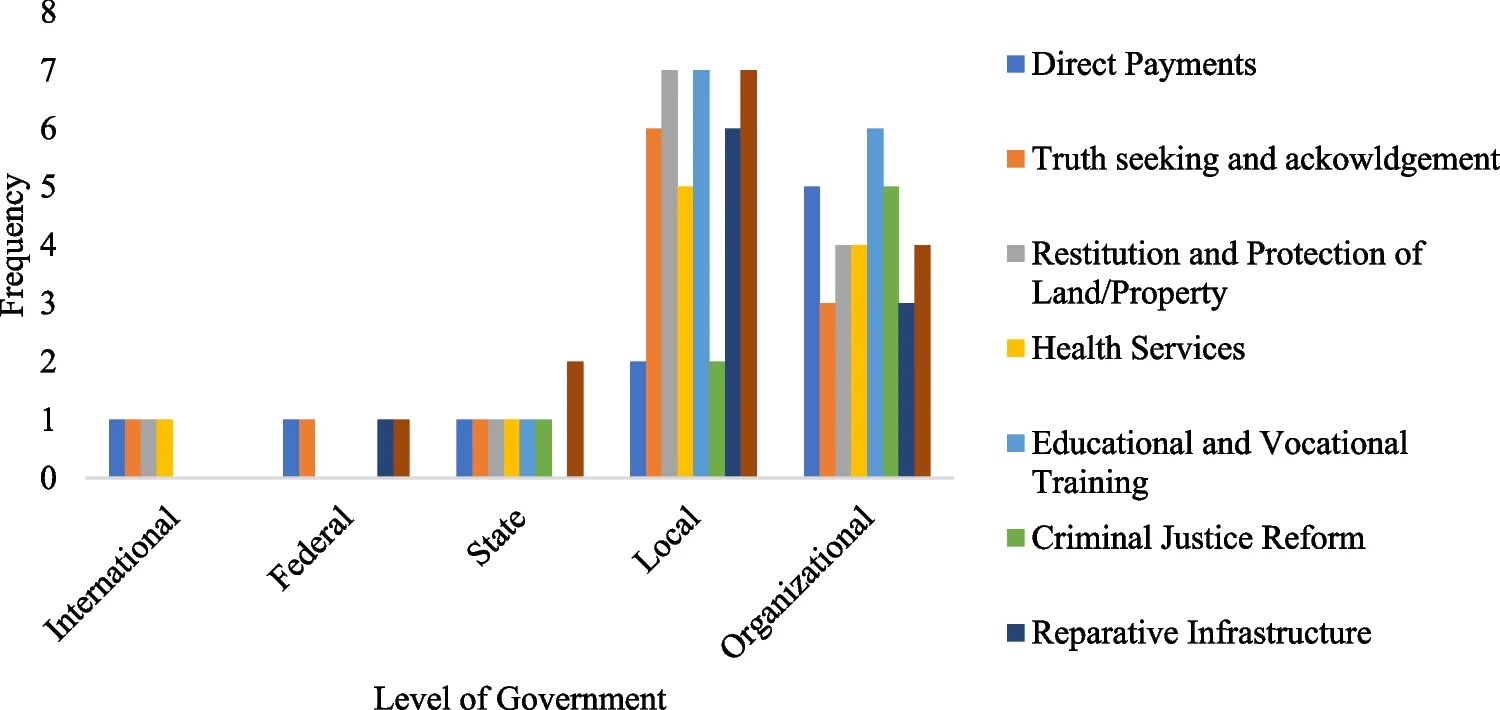
Types of reparations recommended by level of government. Note: H.R. 414 has been reintroduced by the most recent 119th Congress but was only counted once to avoid duplication

Regardless of whether the initiative came from a governmental body or organization, they shared several common recommendations, addressing the third research question (Fig. 3). In addition to many plans expanding reparations beyond ADOS and, in some instances, beyond Black Americans altogether, these initiatives challenge the common conceptualization of reparations as direct payments. More specifically, these initiatives challenge the notion that reparations must come from the *federal* government through direct payments to ADOS.

The recommendations for reparations are extensive, with municipal, state, federal, and organizational nuance often incorporated into governmental plans to address specific regional, national, or institutional transgressions against Black Americans. For example, the Episcopal Diocese of Maryland [81] makes some recommendations based on the church’s role in perpetuating racial injustice. Similarly, the Athens Reparations Action [68] makes recommendations around the city’s urban renewal project, which destroyed a Black neighborhood. These initiatives reflect the core principles of the PHCRP, which emphasizes tackling systemic racism as a root issue and placing reparations within both historical and current contexts [12]. However, for meaningful structural change to occur, there must be improved collaboration between local and national efforts. This coordination is essential to ensure these initiatives work together to break down systemic inequities rather than approaching them in isolation.

#### Subtheme: Truth-Seeking, Acknowledgment, and Apologies

Half of the initiatives analyzed included recommendations for reparations in the form of truth-seeking, acknowledgment, and apologies. These plans seek acknowledgment from white Americans and the governments they collectively control of the historical and ongoing harm to validate their experiences and foster reconciliation. The emphasis on acknowledgment and public apologies underscores the importance of confronting and addressing past wrongs as a crucial step toward healing and justice. For example, the United Nations General Assembly’s recommendations for the International Decade for People of African Descent include truth-seeking and truth-telling to uncover the historical roots of systemic racism [61]. Similarly, the US Congress’s H.R. 414 Reparations Now Initiative calls for a formal acknowledgment and apology from the federal government for state-sanctioned chattel slavery [62]. At the local level, the Austin City Council’s resolution includes an apology for Austin’s participation in slavery and urban renewal projects that decimated Black communities [69].

Organizational initiatives were also vocal in their recommendations and demands for truth-seeking, acknowledgment, and apologies [82]. Coming to the Table includes critical recommendations for white Americans on the individual level, emphasizing the importance of researching, acknowledging, and sharing personal, family, community, state, and national histories of race with openness and honesty [83]. This organization also encourages white Americans to take responsibility for their connections to slavery and to participate in initiatives that support racial justice and reparations.

In evaluating this subtheme via PHCRP, PHCRP acknowledges the significance of truth-telling as an essential component in challenging the dominant narratives put forth by white Americans and institutions that conceal injustices such as structural and systemic racism [12]. Many initiatives not only seek to affirm the lived experiences of Black Americans but also promote reconciliation and ensure systemic accountability. Even so, the PHCRP framework cautions against performative actions that do not lead to tangible change. For example, former Congresswoman Bush’s initiative calls for truth-telling and acknowledgment but stops short of recommending drastic reform such as direct payments or changes to specific pillars of infrastructure. Most initiatives included, however, underscore that enforceable commitments, including reparative education, financial investments, and policy reforms must accompany any public acknowledgments.

#### Subtheme: Financial Compensation and Direct Payments

Though the initiatives extend beyond direct payments or financial compensation as forms of reparations, many also include these components within their plans. This inclusion extends across levels of government and organizational initiatives. For example, the California Reparations Task Force recommends direct payments to Black Californians based on their time in the state, with potential payouts of up to $1.2 million [45]. The Oregon State Legislature’s resolution proposed direct payments totaling $123,000 to eligible applicants, although it ultimately did not pass [65]. The ADOS Advocacy Foundation also advocates for substantial cash payments totaling at least $20 trillion as part of their comprehensive reparations plan [82]. Additionally, the Brookings Institution outlines direct payments, college tuition coverage, and student loan forgiveness as key components to close the racial wealth gap [75]. Financial compensation through direct payments aims to provide immediate and long-term economic relief to descendants of enslaved Africans. By injecting capital directly into Black communities, these payments can potentially help alleviate the economic disadvantages accumulated over generations and allow Black Americans to grow generational wealth.

While immediate financial compensation can offer some relief and signifies the vast amount of debt, the USA owes to Black Americans, PHCRP emphasizes the need for a more profound systemic change to tackle the underlying causes of inequity [12]. Most of the initiatives align with this tenet, framing direct payments as a component that should be a part of a larger strategy that redistributes resources, including housing equity initiatives, investments in Black-owned businesses, and tax structures aimed at repairing wealth extraction practices. This signifies that stakeholders are aware of the limitations a one-time or even staggered cash injection would bring and seek to address socioeconomic and health inequality on a larger and long-term scale.

#### Subtheme: Restitution and Protection of Land and Property

In addition to direct payments, the restitution of land and property was a critical aspect of many reparations initiatives. This is symbolized historically by the unfulfilled promise of “40 acres and a mule” guaranteed to formerly enslaved people after the Civil War [2, 84]. These initiatives make recommendations that would compensate for land unjustly taken from Black Americans as well as protect and commemorate historically significant Black communities and sites. For example, the African Descent-Citizens Reparations Commission [64] in Illinois proposes measures to preserve and grow Black neighborhoods through business development, homeownership, and affordable housing. Similarly, the Linnentown Resolution in Athens, Georgia, addresses the harm caused by urban renewal in the 1960 s by advocating for the return of property and the designation of Linnentown as a historical site [68].

Many initiatives also emphasized the need to protect existing Black communities from further displacement and economic exploitation. This involves creating policies that ensure the sustainability and growth of these communities. The Asheville Community Reparations Commission recommends creating a Land Acquisition Program for future development and establishing financial literacy campaigns to facilitate Black homeownership [67]. Additionally, the African Heritage Reparations Assembly recommends allocating funds for affordable housing and business grants to support the economic stability of Black residents [66].

While initiatives adequately focus on land acquisition and protection, they could go further by integrating policies safeguarding against future adversities, including gentrification and environmental racism, to better align with PCRP [12]. For instance, such initiatives could allocate funding for anti-gentrification strategies, offer legal assistance to uphold property rights, and establish land trusts managed by community stakeholders. Lastly, initiatives should work to address and protect against the harms of environmental racism (e.g., unequal access to green spaces, exposure to lead in water) to ensure a degree of flexibility as required by PHCRP.

#### Subtheme: Health Services, Educational and Vocational Training, and Criminal Justice Reform

Beyond 40 acres and a mule, the reparative initiatives analyzed recommended or demanded reparations in the form of health services, educational opportunities, and vocational training. Concerning health services, systemic racism has contributed to significant health disparities affecting African American communities. Many of the initiatives analyzed sought to address these disparities by funding health services, including medical care, mental health support, and community health centers. For example, the Asheville Community Reparations Commission recommends funding Black-owned and Black-led health centers, establishing healing spaces, and providing payment allocations for years lost due to systemic health inequities [67]. Similarly, the Providence Municipal Reparations Commission includes initiatives to expand health support in disadvantaged neighborhoods and address health disparities [70].

In addition to health and access to quality healthcare, education is a powerful determinant of an individual’s socioeconomic status and overall success [79, 85]. Several reparative initiatives also recommend educational opportunities and equal access to education in their plans to counteract systemic racism in education. This includes the Episcopal Diocese of Maryland’s recommendation for funding and expanding programs specifically designed to support Black children and adolescents’ educational and personal development [81]. Additionally, one of the M4BL’s key recommendations is advocating for free access to public community colleges and universities, offering technical education in fields such as technology, trade, and agriculture [86]. They also support lifelong learning initiatives by implementing educational support programs and retroactively forgiving student loans, promoting continuous learning and advancement. The NAARC advocates for investing in educational programs that empower Black communities and preserve their cultural heritage [31]. Moreover, they propose providing scholarships and educational grants to support African American students’ academic and professional development.

Like traditional education opportunities, vocational training is crucial for providing practical skills and enhancing employment opportunities [85, 87]. In response, the African Descent-Citizens Reparations Commission recommends establishing a Vocational Training Center for people of African descent to promote skilled trade and labor [42, 64]. In Evanston, Illinois, the Equity Empowerment Commission has proposed investing in workforce training programs as part of their reparations plan. These programs aim to enhance African American residents’ job readiness and skills, helping them secure better employment opportunities.

Another area of reform commonly mentioned in the examined reparations initiatives surrounds the criminal legal system. Disparities in the criminal legal system reflect a longstanding and pervasive issue of systemic racism that disproportionately affects Black Americans at every stage, from policing to sentencing and incarceration [88–90]. In response, many of the initiatives analyzed recommended or demanded drastic changes in the form of demilitarizing the police, police training and accountability, ending the school-to-prison pipeline, and protecting the rights of formerly incarcerated inmates. For example, the M4BL advocates for the demilitarization of police as a fundamental step towards ensuring fair and equitable policing practices [86].

Similarly, the Asheville Community Reparations Commission recommends implementing training programs to improve policing practices and reduce racial disparities in law enforcement [67]. The commission also recommends increasing support for restorative justice practices in schools, reducing the presence of police officers on school campuses, and implementing alternative disciplinary measures that keep students engaged in their education. Lastly, the M4BL emphasizes the importance of including formerly incarcerated people in reparations programs to address the lasting consequences of discriminatory sentencing and incarceration practices [91].

This subtheme aligns well with the goals of the PHCRP by tackling structural inequities in health, education, and the criminal justice system [12]. It highlights systemic solutions, such as funding for Black-led health centers, creating vocational training hubs, and increasing educational opportunities, all of which support the PHCRP’s framework in dismantling the root causes of disparities. However, to truly address the challenges faced by Black communities, there needs to be a greater focus on the intersectionality of health disparities, especially for vulnerable subgroups. For example, disparities in Black maternal health illustrate the intersections of race and gender inequities [92]. This necessitates targeted actions like providing culturally competent care, improving access to Black-led birthing centers, and advocating for policy changes to combat systemic biases within healthcare systems.

The rising rates of suicide among Black youth, especially during times of heightened social and economic stress, underline the urgent need for mental health services specifically designed to address their unique pressures [93]. This includes community-based trauma-informed care and mental health support in schools. Moreover, the disproportionate effects of COVID-19 on Black communities reveal the critical need to confront structural inequities that contribute to higher rates of chronic disease, limited access to preventive care, and uneven vaccine distribution [94]. By addressing these intersectional aspects, reparative initiatives can more effectively align with the principles of equity and inclusivity advocated by the PHCRP.

#### Subtheme: Establishment of Reparative Infrastructure Across Different Levels of Government

The last common theme throughout the initiatives is the large-scale establishment of infrastructure that would need to be erected to bring reparations to fruition effectively. Many of these plans, particularly those created by a government body, involve establishing new governing entities and commissions to oversee the reparative process. For example, the California Reparations Task Force recommends establishing the California American Freedman Affairs Agency to administer reparations and support Black Californians in determining eligibility [45]. Similarly, the NAARC, although not government-affiliated, advocates for establishing a National Reparations Trust Authority to manage the distribution of resources and ensure coordinated efforts across various sectors [31]. Local governments, such as Evanston, Illinois, and Asheville, North Carolina, also recommend establishing their own commissions to address specific local needs [42, 67]. These commissions would be tasked with evaluating and implementing programs focusing on housing, economic development, criminal legal reform, and community health​.

The hypothetical establishment of reparative infrastructure presents a unique opportunity to develop systems that confront historical injustices and exemplify egalitarian, anti-capitalist, anti-sexist, and anti-racist frameworks consistent with the principles of the PHCRP [12]. Even so, it remains unclear to what extent Black communities have been or should be engaged in formulating reparation plans. Insufficient community involvement risks perpetuating the power imbalances that reparations seek to address if such infrastructure is one day erected.

To align with PHCRP objectives, the proposed infrastructure must integrate mechanisms for sustained and authentic community engagement, such as participatory decision-making processes [12]. Reparative efforts must include representatives from marginalized subgroups within Black communities. More specifically, women, youth, elders, LGBTQ + individuals, and people with disabilities, thereby ensuring their voices are centered. By adopting such an approach, the governing bodies responsible for overseeing reparations may be able to establish better systems that benefit not only Black men, straight Black Americans, and able-bodied Black Americans but also subpopulations of Black Americans that are the most vulnerable to socioeconomic and health inequality.

### Theme 4: Reparations Initiatives are Influenced by One Another But Largely Uncoordinated

#### Subtheme: Governmental Initiatives are Influenced by One Another but are Less Concerted

The fourth theme and final research question surrounded how coordinated modern reparative efforts were. As evidenced in Table 4, while some are designed to support others, the push for reparations is less than concerted. This means that while one body may use another initiative for reference, they are not necessarily coordinating with one another to propel the reparations movement forward outside of their respective state or municipality. There is, however, some degree of vertical coordination, where federal initiatives, such as H.R. 40 and H.R. 414, have influenced state and local reparations efforts by directly citing the federal bills in their plans [46, 60]. Many local and state initiatives have modeled their efforts after the exploratory commission model proposed in H.R. 40 and H.R. 414. For example, California’s and Evanston’s reparations task forces have taken inspiration from federal guidelines [42, 45].

Many governmental and organizational initiatives, such as the NAARC, follow what Reneau calls the commission model [32, 50]. This approach has become prevalent in reparations initiatives at various governmental levels, starting with Asheville, North Carolina [67]. It is considerably in line with PHCRP and typically involves establishing a commission or task force to study the impact of historical injustices, particularly slavery and systemic racism, and recommend reparations.

These commissions are primarily research-oriented, gathering data, conducting hearings, engaging with affected communities, and reviewing existing literature to develop comprehensive proposals [50]. They usually comprise scholars, community leaders, legal experts, and sometimes government officials, operating within a defined timeframe to complete their work and issue a final report. Evanston, Illinois, is a successful example of the commission model [41]. This model has been widely cited as a successful example of local reparations efforts and has influenced other initiatives; however, these commissions are largely siloed with little coordination between them.

#### Subtheme: Reparations Initiatives are Fraught with Challenges

**Fig. 4:**
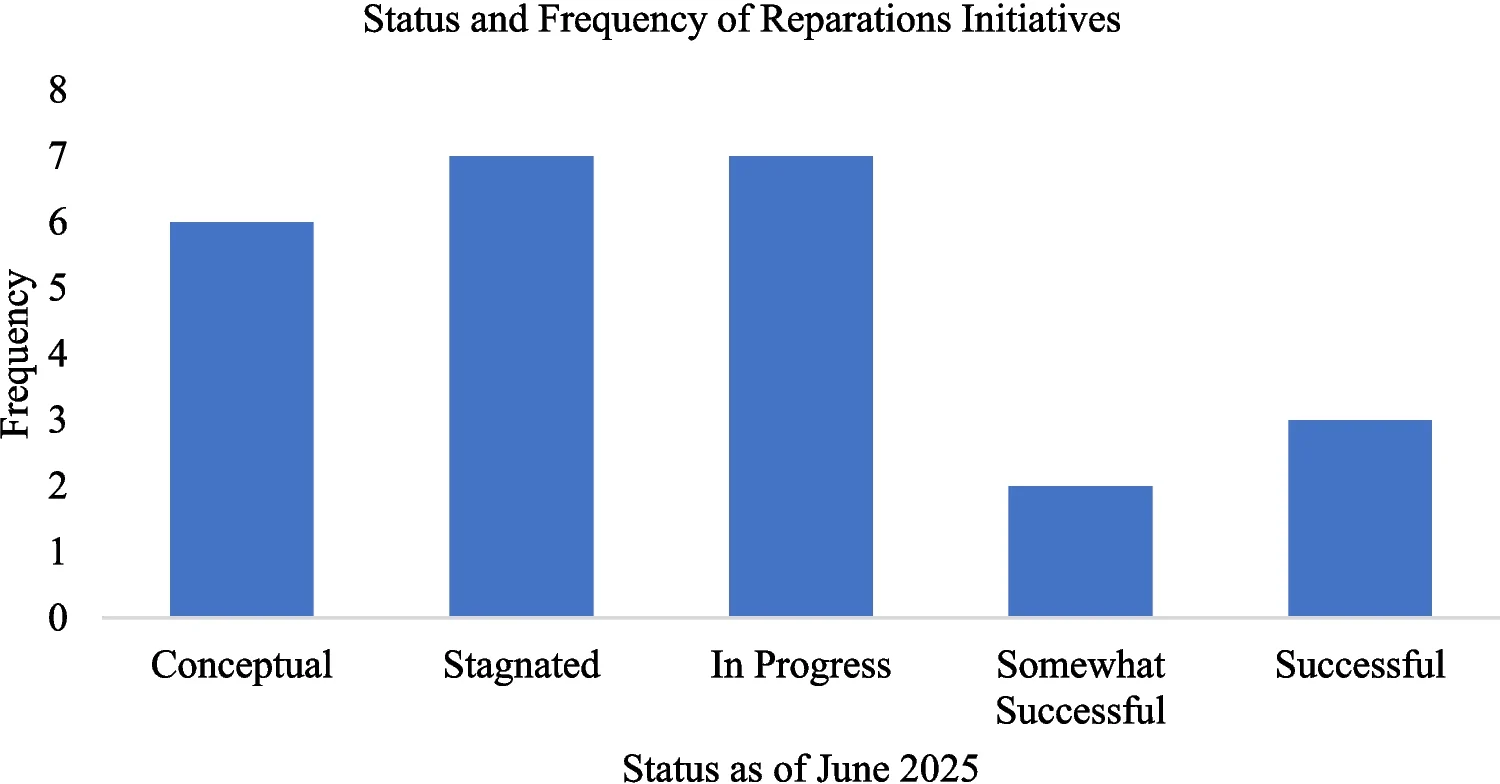
Current status of reparative initiatives as of June 2025

In addition to the lack of coordination between and within governments and organizations, the fight for reparations is plagued by other substantial challenges that have significantly limited its success in most capacities (Fig. 4) [50]. Political resistance remains a formidable obstacle, as reparations are a contentious issue, and significant opposition from conservative political factions can slow legislative progress and reduce the allocation of necessary resources. Secondly, public perception also presents a challenge, with white Americans primarily opposed to reparations [34]. The need to engage and educate the broader public aligns with PHCRP’s goal of fostering critical consciousness to challenge dominant narratives and mobilize collective action.

Barriers are also inherent to specific forms of government. Table 5 outlines the obstacles to initiatives that have stagnated, with lack of political support being a common issue among all initiatives. Local initiatives, like St. Paul’s, rely on limited funding streams, while federal efforts, such as H.R. 414, face legislative delays and lack of political support, leaving local governments to act independently [46, 74]. This contributes to a fragmented movement, with state, regional, and organizational stakeholders waiting for more powerful actors, such as Congress and the president, to act, and such powerful actors are limited within themselves.

**Table 5:** Barriers indicating why specific reparative initiatives have stagnated

Overseeing body	Funding	Lack of political support	Constituent backlash
Athens Reparations Action	X	X	
Black Austin Coalition	X	X	X
California Reparations Task Force		X	X
Oregon General Assembly		X	
San Francisco African American Reparations Committee	X	X	
St. Paul Reparations Legislative Advisory Committee	X	X	
118th Congress		X	X

Though H.R. 414 stagnated during the 118th Congress, it has been reintroduced by the 119th Congress in January of 2025

## Discussion

Using PHCRP as an evaluative framework, this rapid review compares the existing reparations initiatives in the United States and evaluates their effectiveness in mitigating socioeconomic and health disparities affecting Black Americans [12]. Four key themes emerged: ADOS should, at minimum, be eligible for reparations; governmental and organizational initiatives differ in goals; reparations extend beyond direct payments; and reparations initiatives are largely uncoordinated.

Regarding the first theme, the eligibility criteria for reparations differ from contemporary discourse, which focuses on reparations for African Descendants of Slavery (ADOS) [1, 3]. Considering non-ADOS for reparations could reflect changing sentiments regarding slavery, particularly as more Black people, regardless of ethnicity, are impacted by anti-Blackness in an increasingly globalized world. As this conversation evolves from reparations for ADOS (i.e., an ethnic group) to reparations for Black people in America (i.e., a racial group), however, additional barriers may be encountered as white Americans push for colorblind policies (e.g., the latest ruling on Affirmative Action) [95].

More importantly, there are significant socioeconomic and health disparities among ADOS compared to other Black ethnic groups, particularly more recent immigrants [96]. For example, Black Americans tend to be less educated and have higher rates of chronic health conditions compared to those who recently immigrated from African or Caribbean countries [97]. These disparities are worse among Black groups (e.g., women, older Black adults) who exist at the intersection of race and another marginalized identity (e.g., gender, age) [98]. Demonstrably, Black women are three times more likely to die in childbirth than white women, Black men who have sex with men (MSM) are five to ten times more likely to contract HIV than straight Black men, and older Black Americans are likely to die four years sooner than their white counterparts [77, 96, 97].

In reparative discourse, this could pan out to be less than favorable for Black Americans or ADOS, despite the topic of reparations originating around the needs of these groups. Reparative policies must balance inclusivity with acknowledging the distinct historical and structural harms faced by ADOS, as emphasized by PHCRP’s rejection of colorblind and generalized approaches.

Regarding the second theme, governmental initiatives focus on incremental changes within existing systems, such as housing, policing, and economic opportunities, while organizational initiatives, including those from the NAARC and the M4BL, advocate for more structural changes [31, 32]. This may be because governmental initiatives focus on what is in *their* control with the understanding that their efforts are more likely to be brought to fruition or scrutinized by constituents. Conversely, organizations have more flexibility in what they can demand from policymakers. Ultimately, this raises concerns that governmental initiatives will be limited in their ability to truly address socioeconomic or health inequalities, as their strategies are more reactive than proactive. As proponents of PHCRP advocate for dismantling entrenched power dynamics, stakeholders within all levels of government should focus on redistributing power and resources to Black communities, actively working to build consensus and bring opposition on board, and facilitating reparative efforts instead of relying on safe, soft language and incremental approaches that fail to address systemic inequities at their core.

The third theme surrounded extending reparations beyond direct payments. Like the eligibility requirements commonly conceptualized in the discourse around reparations, the initiatives examined argue that reparations should include more infrastructural changes than direct payments. This theme represents two possibly conflicting but popular stances: (a) that addressing socioeconomic and health inequality among Black Americans requires a comprehensive effort beyond what money can solve, and (b) that Black Americans do not have enough financial literacy to use the money given to them to address their own hardship. Instead, they would transfer the funds back into retail and consumer industries by spending money on material items.

Darity, Lahiri, and Frank refer to this as a “transfer problem” [98]. The former speaks to the all-encompassing nature of inequality. At the same time, the latter reflects the long-held racist idea that Black Americans are incapable of socioeconomic improvement without the overseeing of white Americans. In line with PHCRP, stakeholders should examine their paternalistic assumptions regarding Black Americans’ capacity to make informed decisions while considering the limitations of direct payments in addressing socioeconomic and health inequality.

The fourth theme highlighted that reparations initiatives influence each other but are largely uncoordinated. While the discourse usually implicates the federal government as the entity responsible for paying reparations, most initiatives are carried out at local, state, and organizational levels. This reflects the increasing interest of smaller entities, who prefer not to wait and distrust the federal government to act. These smaller state, local, and organizational stakeholders might also be more capable of addressing their constituents’ socioeconomic and health needs than state or federal governments. However, this decentralized approach restricts the ability to hold white institutions, corporations, and individuals accountable, which PHCRP emphasizes as vital for dismantling systemic inequities. Improved coordination among local, state, and organizational initiatives could encourage collective progress.

### Policy Implications

#### Reparations from the Bottom up, not the Top Down

When considering the numerous reparative initiatives taking place at the state and local levels, several policy suggestions arise for local stakeholders in the fight for reparations. First, reframing discussions about reparations from a windfall granted to ADOS by the federal government to a practical option that local and state governments, along with organizations, can pursue both independently and collaboratively. Given the greater likelihood of successful collaboration among communities as opposed to states, municipalities could act as the starting point for expanding the reach of reparations initiatives. Promoting consistent collaboration among municipalities could facilitate the sharing of best practices and resources, creating a unified approach that respects local specificity.

Local collaboration is vital for more liberal municipalities in conservative states such as Shelby County, Tennessee [72]. As these initiatives stand, a significant challenge faced by both governmental and organizational initiatives is their limited ability to hold white institutions, white corporations, and white Americans accountable. A pipeline must be created for poorer and even middle-class white Americans that flows between achieving class consciousness and embracing anti-racism. One potential strategy could be targeting liberal towns and cities within conservative states and finding ways to introduce and build reparative initiatives in these liberal enclaves. By building class consciousness (i.e., the awareness among poorer white Americans that they, too, are being economically exploited like people of color), such efforts could serve as blueprints for neighboring areas and potentially spark broader regional change [99].

Another recommendation is to reframe reparations as a public health issue rather than viewing it solely as a socioeconomic initiative that requires direct payments. By framing reparations in this manner, states and municipalities can more effectively address health disparities and improve the quality of life for Black Americans in a more immediate and localized way. Additionally, reparations should encompass more than just direct financial payments; they must also address social determinants of health, including land restitution, criminal justice reform, and access to education, vocational training, and healthcare services. A comprehensive approach to reparations, regardless of political ideology, is crucial to promote lasting change and confront the root inequalities faced by Black communities.

#### Intergovernmental Coordination

As discussed in themes three and four, there is a strong consensus that reparative infrastructure (e.g., a National Reparations Commission) is essential in the fight for reparations. Still, initiatives often lack intergovernmental coordination to implement plans and policies on a large scale. Several strategies could be employed to address this. First, establishing intergovernmental task forces (e.g., incorporating state and local policymakers) and forming coalitions among stakeholders could align resources, amplify successful models like Evanston’s, and elevate marginalized voices in line with the advocacy of the PHCRP [12]. Second, implementing shared eligibility criteria could standardize frameworks and streamline funding distribution, promoting equity and expanding outreach. One approach could include granting reparations eligibility to all Black Americans while offering additional reparative support (e.g., higher direct payments) to ADOS. Third, creating collaborative platforms, such as a federally run website dedicated to knowledge sharing that includes case studies and best practices, would encourage the adaptation of proven models. Ultimately, drawing on international initiatives, such as those led by South Africa’s Truth and Reconciliation Commission, can offer valuable guidance for US initiatives [100].

#### Social Liberalism and Progressivism Might Temper Black Radical Thought

Socially liberal and progressive viewpoints were apparent in both governmental and organizational initiatives [59]. Therefore, a key policy implication is the need for reparative advocates to remain mindful of the Black radical messages of resistance and liberation that have sparked social movements from slave rebellions to the Black Lives Matter Movement [2, 37, 101]. Rather than proposing revolution, the reviewed plans favor reforms, as opposed to a broader revolution, and limit the ability of reparations to address socioeconomic or health inequality. The reviewed initiatives attempt to reform currently existing infrastructure, such as healthcare and the carceral system, that is principled by mutually reinforcing racism and capitalistic exploitation [99, 102]. Any reform, instead of a revolutionary reparative movement, feeds resources back into inherently racist systems instead of contributing to their dismantling.

### Limitations

The limitations of this rapid review mainly relate to the scope, depth, and breadth of the analysis. First, rapid reviews inherently sacrifice a more thorough exploration of available data due to time constraints, which can result in the omission of significant studies or nuances within the reparations discourse [52]. This limitation can lead to gaps in understanding ongoing initiatives or their long-term effects. Another limitation of this study is the absence of inter-coder reliability testing, as all qualitative coding was conducted by a single researcher. Although reflexive memoing was employed to enhance transparency and reduce interpretive bias, the lack of multiple coders may limit the generalizability and reproducibility of the findings. Lastly, this review is predominantly qualitative in nature, which restricts its ability to quantify the impact of reparations initiatives or to provide statistical comparisons across efforts.

## Conclusion

In conclusion, this rapid review highlights the complexity and evolving nature of reparations initiatives for Black Americans in the USA. The findings suggest that meaningful reparative justice requires an alignment with PHCRP and moving beyond liberal and progressive reforms to challenge the systemic roots of racism and capitalistic exploitation [12]. Fostering collaboration across municipalities, reframing reparations as a public health issue, and emphasizing class consciousness are crucial strategies for advancing these initiatives that are currently not being implemented. While progress has been made, the pursuit of reparations demands sustained effort, innovative approaches, and a commitment to addressing the deep-rooted inequities that continue to affect Black Americans.

More importantly, in the current political landscape, marked by the reinstated Trump administration, reparative progress faces grave threats [103]. Yet this regression may serve as a catalyst. As the USA moves further right, the intensifying weight of oppression could ignite a reparative revolution. This may, in turn, push advocates toward bolder, more radical strategies that resist and overthrow white supremacy instead of operating within the bounds of liberalism.

## Data Availability

Data included in this review is available upon request.

## Appendix 1

Table 6

**Table 6:** Table outlining the details and recommendations of the initiatives analyzed

International initiatives									
**Organizing body**	**Name**	**Location**	**Eligibility criteria**		**Description**				**Status as of 2025**
**United Nations General Assembly** **(2016)**	Implementation of the International Decade for People of African Descent	Geneva, Switzerland; New York, New York	African people and people of African descent, including those in the U.S		An operational framework countries can use to address the ongoing consequences of enslavement, the transatlantic slave trade, colonialism, apartheid, genocide, and past tragedies for people of African descent				The US Department of State released a statement in support of the plan in 2021. In 2025, the African Union designated the year as the “Year of Reparations: Justice for Africans and People of African Descent.”
	**Recommendations to International Members of the U.N** 1. Truth-seeking and truth-telling surrounding the historical roots of systemic racism 2. A public apology and acknowledgment of the current and historical injustices 3. Memorialization including paying tribute to victims and preserving historical sites 4. Compensation for economic disenfranchisement 5. Restitution of land, cultural heritage, natural resources; Repatriation for ADOS 6. Rehabilitation to address physical and psychosocial health consequences 7. Guarantees of non-repetition of future violence and oppression								
**Federal initiatives**									
**Organizing body**	**Name**	**Location**	**Eligibility criteria**		**Description**				**Status as of 2025**
**US 118th & 119th Congresses (2023, 2025)**	H.R. 414 Reparations Now Initiative	Washington, D.C	ADOS		A resolution that aims to advance the momentum around reparations at the federal, state, and local governments for chattel slavery				Introduced a second time to the House Judiciary Committee, where it was referred after its introduction in May 2023
	**Recommendations to Local, State, and Federal Governments** 1. Recognize of the responsibility of the federal government to provide reparations such as financial compensation 2. Pass H.R. 40, which would establish a commission to study and develop reparation proposals for African Americans 3. Issue a formal acknowledgment and apology from the federal government on for state-sanctioned chattel slavery 4. The federal government should acknowledge the momentum legacy organizations, grassroots movements, and national organizations have brought to the modern-day reparations movement 5. Implement state, local, and federal initiatives to identify funding sources for reparations 6. The federal government should honor the lives and legacies of those enslaved and victims of other forms of state-sanctioned violence								
**State initiatives**									
**Organizing body**	**Name**	**Location**	**Eligibility criteria**		**Description**				**Status as of 2025**
**African Descent-Citizens Reparations Commission (ADCRC) (2022)**	20 ILCS 405/405–540	Illinois, U.S	ADOS		This statute establishes the ADCRC, a commission that will recommend measures to preserve and grow Black neighborhoods and communities throughout the state. The recommendations are subject to change				The ADCRC Is currently holding monthly committee meetings as well as public engagement meetings. Commissioners have plans to utilize parts of California’s reparations framework
	**Recommendations to the Illinois State Legislature** 1. Preserve Black American neighborhoods and communities by promoting business development, home ownership, and affordable housing 2. Establish a Vocational Training Center for people of African descent with satellite centers throughout the state. The purpose of this Center is to promote skilled trade and labor 3. Ensure that Black Americans are proportionally represented in the contracts (e.g., vendors) that the state has with private businesses 4. Create and enforce a Slavery Era Disclosure Bill that would negotiate payments between the commission and private corporations or institutions that benefited from slavery								
**California Reparations Task Force (2023)**	California Reparations Report	California, U.S	ADOS in California or Black Californians who can trace their lineage in the USA prior to 1900		The purpose of this report is to address current and historical disparities in health, housing, mass incarceration, housing, etc. citing enslavement, racial terror, and political disenfranchisement. Based on time lived in California, Black Californians could receive a payout up to $1.2 million				The task force issued its final recommendations to the state legislature in July of 2023. Legislative movement stalled but the California Legislative Black Caucus introduced a 2025 legislative package to revisit the issue
	**Recommendations to the California State Legislature** 1. Create and fund the California American Freedman Affairs Agency, which would, amongst other responsibilities, assist Black Californians in determining eligibility and would be tasked with administering reparations in the form of direct payments, education, and legal services 2. Repeal Proposition 209, an amendment to the state constitution that prohibits state institutions (e.g., public universities) from considering race in state contracting practices (e.g., college admissions, loan lending), disparagingly impacting Black students and business owners 3. Identify the racial consequences of laws, policies, and ordinances at both local and state levels before enactment 4. Instruct the Civil Rights Department and the Department of Education to collect anonymized data for all racial complaints, the departments’ responses to the complaints, and to make this information publicly available 5. Declare the state’s interest in rectifying ongoing harm caused by chattel slavery, identify specific harms caused by chattel slavery, and demonstrate that policies are tailored to mitigate specific harms 6. Transmit the findings of the California Reparations Task Force to the US president and Congress								
**Oregon State Legislature (2021)**	Senate Bill 619	Oregon, U.S	ADOS in Oregon, people who identified as African American on legal documents over the last 10 years, has resided in Oregon at least two years before applying, and is over the age of 18		This bill calls upon the Department of Revenue to bring direct payment reparations to ADOS. Though Oregon was not a slave state, the ultimate goals of this bill is to pay restitution for barring Black Americans from the state until 1927				This bill died in the Oregon state center to in June 2021
	**Recommendations to the Department of Revenue** 1. Pay eligible applicants a total pf $123,000 in yearly installments over the life of the applicant								
**Local initiatives**									
**Organizing body**	**Name**	**Location**	**Eligibility Criteria**		**Description**				**Status as of 2025**
**African Heritage Reparations Assembly (2023)**	Final Report	Amherst, MA	Descendants of people who were enslaved in Amherst and elsewhere as well as other people of African descent living in Amherst		This report urges the Town Council to redress racialized harms waged against the town’s Black American residents historically and in the present era				In 2021, the Amherst Town Council approved the creation of a reparations fund. In June 2022, the Council agreed to transfer up to $205,000, increasing to $2 million over 10 years. The assembly issued the final report to the Town Council of Amherst in of September 2023
	**Key Recommendations to the Amherst Town Council*** 1. Operationalize a $2 Million Reparations Endowment Fund Within 4 Years from cannabis sales tax 2. Collaborate with other Municipalities to advocate for statewide legislation 3. Allocate funds to three primary domains: youth programming; affordable housing; and business grants & entrepreneurial training 4. Continue and Expand Town-Wide Programming in Truth and Reconciliation 5. Publicize the Human Rights Commission’s complaint process and consider a town policy for public apology 6. Provide assistance to residents for the expungement of cannabis charges 7. Provide resources to the board of health to address health inequities								
**Asheville Community Reparations Commission (2020)**	Community Reparations Commission Draft Recommendations	Asheville, NC	To be fully determined but the initial draft includes Black Americans living in Asheville		This plan makes short-, medium-, and long-term goals to address systemic racism, citing slavery, segregation, and ongoing racial atrocities throughout the state				The Commission completed its recommendations in 2024, passing 39 policies aimed at addressing systemic racism. In 2025, meetings continue to discuss implementation strategies
	**Preliminary Recommendations to the City Council of Asheville** 1. Address racial disparities within the criminal legal system and promote equity and accountability. This includes implementing training programs to improve policing and allocating funds to support and creating initiatives to break the school-to-prison pipeline 2. Establish an Economic Development Center, small business incubators, and Black business corridors, as well as the provision of grants to support Black-owned businesses 3. Address educational disparities and promote equity for Black youth in Asheville and Buncombe County. This includes establishing community-based education centers, providing enrichment experiences, and supporting educational international travel 4. Address health-related disparities and promote equity by incorporating payment allocations for years lost, funding Black-owned and Black-led health centers, and establishing healing spaces, including birthing centers and healing circles 5. Create a Land Acquisition Program for future development, establishing a Dollar Lot Program to allocate reparations land for aspiring Black homeowners, and implement financial literacy and action campaigns to facilitate Black homeownership								
**Athens Reparations Action (2021)**	The Linnentown Resolution	Athens, GA	Descendants of individuals who lived in Linnentown whose homes were destroyed for urban renewal		This resolution recognizes and redresses the harms caused by urban renewal in the 1960 s wherein Black communities were destroyed and property was seized. The goal is to work towards improving neighborhood infrastructure (e.g., affordable housing, transportation)				The City Council has asked for $5 million towards the initiative. Athens-Clarke County government has pledged 2.5 million dollars but the University System of Georgia, who was asked to pay the other half, has refused
	**Recommendations to the Athens-Clarke County Unified Government** 1. Acknowledge the injustice and harm to Linnentown and other Black communities as a result of urban renewal 2. Partner with the University System of Georgia (USG) will recognize the legacy of Linnentown through an on-site memorial 3. Determine the amount of intergenerational wealth lost to urban renewal and make budgetary accommodations for affordable housing, economic development, telecommunication, public transportation, and public art 4. Designate Linnentown as a historical site, erect historical placards, and register the National Register of Historic Places 5. Partner with the USG to create and fund a Center on Slavery, Jim Crow, and the Future of Athens Black Communities 6. Examine policies regulating property acquisitions via land swaps between the UGA and USG 7. Urge the Georgia General Assembly to establish an Authority on Recognition & Redress to acknowledge the harm undone to Black communities through slavery, Jim Crow, redlining, and urban renewal and to determine appropriate forms of compensation 8. Deliver copies of the resolution to Georgia state lawmakers								
**Black Austin Coalition (2021)**	Resolution No. 20210304–067	Austin, TX	To be fully determined but the resolution currently includes Black residents in Austin		The purpose of this resolution is to create an economic model of reparations for Black Austinites. The goal of this resolution is to provide restitution not only to ADOS but for racial injustice perpetuated in Austin in the twentieth century				Progressed stymied in June 2022 due to the city council failing to meet the deadline for a study on reparations in Austin
	**Recommendations to the Austin City Council** 1. Apologize for Austin’s participation in slavery, racial segregation, exacerbating racial divides, and carrying out urban renewal projects that decimated Black communities 2. Reject prejudice based on race, religion, gender, sexual orientation, or nationality 3. Commit to correct the city’s systemically racist practices and rectify the effects of those practices 4. Urge the county, school district, state of Texas and federal government to provide reparations at all levels of government 5. Conduct a study and produce a report that outlines the economic value of racial harm perpetuated by Austin with the assistance of universities in Texas 6. Create a centralized Black Embassy, or a culture center geared toward the success of Black owned businesses and organizations in Austin 7. Include stakeholders in the planning of the creation of the Embassy such as Black businesses, communities, and residents								
**Equity Empowerment Commission (2019)**	Recommendations on actions to address wealth and opportunity gaps	Evanston, IL	Black residents and their direct descendants that lived in Evanston between 1919–1969			This memorandum makes recommendations to revitalize, preserve, and stabilize Black American ownership and generational wealth in Evanston. This initiative addresses discriminatory housing practices when they were most prevalent before 1969			As of June 2025, the Local Reparations Restorative Housing Program in Evanston, Illinois, has allocated reparations funds totaling over $5 million. However, the city is now being sued by Judicial Watch, a conservative legal group arguing that the bill is racially discriminatory against non-Black residents
	**Recommendations to the Evanston City Council** 1. Provide property tax relief to African American, long-time owners of residential property in Evanston 2. Provide housing repair and rehabilitation assistance to African American property owners in Evanston 3. Provide down payment assistance to income-qualified, African American home purchasers in Evanston 4. Provide housing rental assistance to income-qualified, African American residents in Evanston 5. Re-purpose the Gibbs-Morrison Center to provide co-working or work cooperative space for African American entrepreneurs 6. Invest in workforce training for African American Evanston residents 7. Provide low-interest loans for African American Evanston entrepreneurs								
**Providence Municipal Reparations Commission (2022)**	Report of the Providence Municipal Reparations Commission	Providence, RI	People living in Providence who have African or Indigenous heritage or are earning less than 30% of the area median income			This final report makes recommendations help reduce the racial wealth gap for city’s Indigenous and African heritage populations and their neighborhoods. Providence’s role in upholding and benefitting from slavery, racial segregation, and ongoing racial discrimination are grounds for reparations			In November 2022, the mayor allocated $10 million towards of reparations fund. As of August 2024, over $1.5 million of those funds have been distributed to Black organizations, not citizens, to use at their discretion. In December of 2024, over $4 million of those funds were allocated elsewhere in the city’s budget
	**Recommendations to the Providence City Council** 1. Recognize the harm done to Indigenous and African heritage people in the form of apologies for slavery, land theft, urban renewal practices, and the criminal legal system 2. Adopt several programs that simultaneously build wealth and financial stability such as a Home Ownership and Financial Literacy Program, a Guaranteed Income Program, and a Earn and Learn Workforce Program 3. Create and develop African heritage and Indigenous media, technology, and communication companies 4. Create African and Indigenous heritage programs such as grants for businesses, programs that preserve and promote their culture, and programs that support community health and criminal justice reform 5. Review and reform laws and policies that harm African heritage and Indigenous people and communities 6. Move towards a more equitable healthcare system by expanding health supports in disadvantaged neighborhoods, expanding health provider engagement with local schools, and collaborating with community partners to reduce health disparities 7. Develop neighborhood-based incubators that serve the direct socioeconomic needs of residents of Indigenous and African Heritage, 8. Expand the African American Ambassadors Group that has proactively addressed racial, social, and economic equity gaps for African heritage residents 9. Create an “African heritage and Indigenous survivors & descendants of providence urban renewal displacement” fund 10. Expand representation of African heritage and Indigenous people in governing bodies								
**San Francisco African American Reparations Committee (2023)**	San Francisco Reparations Plan 2023	San Francisco, CA	ADOS or descendants of free Black people prior to the end of the nineteenth century. Applicants must be adults, have identified as Black/African American on public documents, born or moved to San Francisco prior to 2006, and has lived in the city for at least 10 years		This final report is designed to improve the education, housing, workforce development, financial stability, and other areas of inequality for Black San Franciscans. While California was not a slave state, San Francisco participated and continues to participate in the displacement, disenfranchisement, and segregation of its Black inhabitants				As of December 2023, the mayor of San Francisco has cut the $4 million that was due to be allocated to a potential reparations office that would oversee this plan. The mayor further argued that reparations should be addressed at the federal level, not at the local level
	**Key Recommendations to the City and County of San Francisco*** 1. Apologize for past harms and commit to making substantial ongoing, systemic and programmatic investments in Black communities to address historical harms 2. Establish an independent Office of Reparations within the City to execute this plan 3. Create and fund a committee of community stakeholders to ensure equity and continuity in the following areas: housing, employment, education, healthcare, and overall policy 4. Create and sustain thriving, complete neighborhoods that include commercial activity, open spaces, safe streets and affordable housing for Black San Franciscans 5. Align educational, professional, and economic development pathways to ensure successful outcomes across all employment levels 6. Invest in educational infrastructure to ensure that all students have equitable access to quality school buildings and resources 7. Offer creative, community-informed options to support students who are most at risk of becoming involved in the school-to-prison pipeline 8. Address and reduce health disparities by investing in structural, long-term solutions to the social determinants to health 9. Address the historical and existing state policies that have disproportionately harmed San Francisco’s African American communities 10. Enforce existing local policies that are ostensibly designed to address historical harms								
**Shelby County Commission Reparations Committee (2023)**	Shelby County Reparations Resolution	Shelby County, TN			To be fully determined following the final recommendations of a reparations plan		This resolution provides preliminary recommendations prior to a feasibility study that would make further refine these recommendations. The resolution cites previous harms to African American communities in Shelby County by addressing poverty and barriers to economic mobility		As of February 2023, the Shelby County Commission approved $5 million towards a reparations program, starting with the implementation of a feasibility study. The initiative has stagnated since this approval
	**Preliminary Recommendations to the Shelby County Commission** 1. Increase access to affordable housing, African American homeownership, and receiverships while providing education for the consumer and combating practices that hinder African American communities 2. Identify critical needs within the mental health realm and discovering ways to provide affordable healthcare in areas of socioeconomic need 3. Identify areas within the criminal justice system that have shown historical and systemic disenfranchisement of African Americans and providing holistic solutions 4. Enhance career opportunities and business ownership, while promoting higher pay to close the wealth gap among African Americans and other demographics 5. Create short, medium, and long-term strategies to challenge and confront negative elements such as predatory lending while promoting financial literacy, financial freedom, and generational wealth								
**South Bend Common Council Reparatory Justice Commission (2023)**	Bill No. 22–61 A Resolution Calling for Reparatory Justice	South Bend, ID			To be fully determined following the final recommendations of a reparations plan		The purpose of this resolution is to urge the city council provide retribution for African American for past discrimination and to prevent future racial injustice. The ultimate goal is to reform structural mechanisms in healthcare, employment, and other areas		As of June 2025, the Reparatory Justice Commission continues to hold public meetings on the first Tuesday of each month
	**Preliminary Recommendations to the South Bend City Council** 1. Apologize for past discrimination and harm incurred by past practices 2. Establish a Truth and Reconciliation Commission to advice the city on how to make financial reparations to African Americans 3. Invest in institutions and programs that provide affordable housing, psychological counseling, health care, and vocational training 4. Compel the South Bend Community School Corporation to work with the city to incorporate reparative justice and African American history into its curriculum and history lessons 5. Support the creation of public memorials that honor African American history in the city 6. Set aside a significant portion of federal American Rescue Plan and Infrastructure Investment and Jobs Act dollars to fund reparative programs								
**St. Paul Reparations Legislative Advisory Committee (2022)**	Resolution 21–77	St. Paul, MN			ADOS		This resolution acknowledges the historical and ongoing impacts of slavery and systemic racism on African Americans, and it aims to address these issues through several specific measures		The commission is actively working on recommendations to promote generational wealth and economic mobility for Black residents, with ongoing community engagement and support from city officials
	**Recommendations to the City of St. Paul** 1. Apologize and commits to rectifying its involvement in institutional racism against the descendants of chattel slavery 2. Apologize and commit to rectifying the wrongful bondage of Dred Scott at Fort Snelling and the city’s enforcement of institutional racism and related discriminatory practices 3. Apologize and commit to addressing the destruction of the vibrant Black community and successful Black businesses in the Rondo neighborhood due to the construction of Interstate 94 4. Urge all local organizations and institutions that have benefited from racial inequity to join the city in these apologies and to address racism within their own operations, working collaboratively with the city to address systemic racism comprehensively 5. Call on the State of Minnesota and federal representatives to begin policymaking and secure funding for reparations at both state and national levels 6. Establish a legislative advisory committee to define the roles and responsibilities for a new commission named the Saint Paul Recovery Act Community Reparations Commission 7. Make short, medium, and long-term recommendations to specifically promote the creation of generational wealth and enhance economic mobility for the descendants of chattel slavery in the Black community 8. Issue a report for the city’s consideration, focusing on strategies to increase equity and generational wealth, and close disparities in homeownership, healthcare, education, employment, etc								
**Organizational initiatives**									
**Organizing body**	**Name**	**Location**			**Eligibility criteria**		**Description**		**Status as of 2025**
**American Descendants of Slavery (ADOS) Advocacy Foundation (2016)**	ADOS Reparations Agenda	Decatur, Georgia			ADOS		ADOS’ plan seeks comprehensive reparations and the establishment of an office dedicated to addressing the specific needs and historical injustices faced by the descendants of chattel slavery in the United States		In the Spring 2024, ADOS extended their launch of local chapters across the USA. The foundation meets at a yearly summit to coordinate around issues impacting ADOS, including the reparations
	**Key Recommendations to the US Government*** 1. Administer a minimum of $20 trillion in cash payments to be distributed to eligible descendants of enslaved Africans in the United States 2. Designate descendants of US chattel slavery as a protected category, ensuring targeted policies and protections 3. Establish a Federal Office of ADOS Affairs to manage the reparations program, assist in verifying eligibility through genealogical research 4. Other reparative measures including land allocations, educational opportunities, mental health services, and legal protections								
**Brookings Institution (2020)**	Why we need Reparations for Black Americans	Washington, DC			ADOS		The plan aims to restore lost wealth, provide economic opportunities, and redress systemic inequities stemming from slavery and discriminatory policies		As of now, the plan for reparations outlined remains a proposal
	**Recommendations to the US Government** 1. Administer direct payments close the racial wealth gap by compensating descendants of enslaved Black Americans 2. Provide college tuition coverage and student loan forgiveness for descendants of enslaved Black Americans 3. Offer down payment grants and housing revitalization grants to promote homeownership and improve housing conditions in Black communities 4. Supporting Black-owned businesses with grants for startup, expansion, and property purchases								
**Coming to the Table (CTTT) (2021)**	A Guide to the Reparations Movement	Oakland, California			ADOS		Their guide outlines a comprehensive approach to addressing and rectifying the historical injustices inflicted upon African Americans due to slavery, Jim Crow laws, and ongoing systemic racism, particularly at an interpersonal level		Coming to the Table has over 40 local affiliate organizations across the USA in different stages of reparative work. The continue to host annual meetings
	**Key Recommendations to White Americans *** 1. Research, acknowledge, and share personal, family, community, state, and national histories of race with openness and honesty 2. Connect with others across racial lines in order to develop and deepen relationships 3. Heal racial wounds by white Americans, among other things, taking responsibility for their connection to slavery, acknowledging their own personal racial transgressions, research their familial link to slavery, and participating in Coming to the Table’s affiliate groups 4. Take action by, amongst other things, joining or donating to other racial justice organizations, speaking out in support of laws that support racial justice, finding ways to support African Americans through public service, and patronizing African American owned businesses 5. Advocate that local, state, and federal governments administer reparative payments to African Americans by supporting reparations initiatives, voting, and holding lawmakers accountable 6. Push for criminal justice reform. Namely, the demilitarization of the police force, increased training for the police force, and ensuring formerly incarcerated people are eligible for reparations 7. Promote economic justice by, amongst other things, creating mechanisms for reclaiming land that was taken by African Americans, creating jobs and opportunities for Black Americans, and develop a money management system to manage direct reparations payments 8. Facilitate equal access to quality education by, amongst other things, working with educators to reform Eurocentric curricula that includes the full impact of slavery on the U.S., creating scholarships for African Americans, supporting research on racial conflict resolution, and establish mentorship programs for white Americans 9. Promote community by, among other things, expanding Coming to the Table Affiliate Groups in local communities across the U.S., recreating African American Green Books that identify African American owned businesses, and advocating for food justice 10. Reduce racial and ethnic health disparities by, among other things, increasing access to quality health care, updating the Diagnostic and Statistical Manual of Mental Disorders to include racial trauma, and encouraging mental health providers to provide culturally conscious care 11. Preserve African American history by, among other things, by donating familial materials regarding ancestral connections to slavery, establishing a national genealogical database, and advocate for markers, plaques, museums acknowledging historical events related to African Americans								
**Decolonizing Wealth Project (DWP) (2018)**	Seven Steps to Healing	New York, New York	Indigenous communities and communities of color that have been historically marginalized and impacted by colonization, including ADOS		Decolonizing Wealth Project outlines a framework of seven steps to healing. These steps are designed to address and heal the wounds caused by colonization and systemic inequities within the philanthropic and financial sectors				In June 2023, DWP pledged to $20 million over a span of five years to reparations initiatives across the USA. In June 2025, DWP unveiled its ambitious “Moonshot” strategy, a 10-year plan to catalyze $1 trillion in reparative giving by 2035
	**Recommendations to American Individuals, Organizations, and Organizational Actors** 1. **Grieve:** Acknowledge and mourn the pain and trauma caused by colonization and systemic injustice 2. **Apologize:** Offer sincere apologies for the wrongs committed and the suffering inflicted on marginalized communities Engage in deep, empathetic listening to the voices and experiences of those who have been harmed 3. **Listen:** Build authentic relationships based on trust, respect, and mutual understanding 4. **Relate:** Ensure that marginalized communities have a seat at the table and are meaningfully represented in decision-making processes 5. **Represent:** Create entirely new decision-making structures rather than merely adding token positions to existing, colonial frameworks as an afterthought 6. **Redistribute:** Redistribute resources and wealth to support the self-determination and empowerment of marginalized communities 7. **Repair:** Take concrete actions to repair the damage caused by colonization and systemic inequities								
**The Civil Rights and Restorative Justice Project (2023)**	A Toolkit for Policymakers and Advocates	Boston, MA	Individuals who directly experienced racial violence, descendants of survivors who experienced racial violence, and communities, and broader communities that have been affected by systemic racial injustices			The Toolkit serves as a comprehensive guide to help state and local policymakers and advocates develop and implement policies to address and remediate historical racial injustices. It offers both a framework for thinking about reparations and concrete examples of policies that have been adopted across the United States			The Toolkit is actively available as a resource particularly for those at Northeastern University
	**Recommendations to State and Local Policy Makers and Organizational Actors** 1. **Restitution**: Restore victims to their original state before the violation occurred. This may involve addressing the structural causes that led to the violation, such as systemic discrimination 2. **Compensation**: Compensation should be adequate to cover all harms suffered, including medical and legal costs, as well as the loss of educational and employment opportunities 3. **Rehabilitation**: Rehabilitation should be comprehensive, offering medical and psychological care, legal services, and social support 4. **Satisfaction and the Right to Truth**: This involves the verification and public disclosure of facts related to the violation, identifying victims, issuing public apologies that acknowledge and take responsibility for the violations, and commemorating the victims 5. **Guarantees of Non-Repetition**: Measures should be implemented to prevent future violations, including changing relevant laws and putting in place preventative and deterrent measures								
**The Episcopal Diocese of Maryland (2019)**	Resolution 2020–06	Baltimore, MD	The church does not explicitly detail who is eligible to receive reparations, aims to repair the historical and ongoing injustices faced by Black and brown people, particularly those affected by slavery			The church emphasizes the importance of reparations as a means of repairing the breaches caused by these injustices and calls on the Church to take actionable steps towards reconciliation and justice			On May 31, 2025, the Diocese awarded $300,000 in reparations grants to six organizations dedicated to uplifting Black communities within Maryland
	**Key Recommendations to Members and Leaders of the Episcopalian Church*** 1. Acknowledge and educate about the church’s and society’s historical and ongoing racial injustices 2. Advocate for a vision of equality and dignity for all, inspired by Christian faith 3. Fund and expand programs to empower Black youth, helping them achieve their potential and avoid the criminal justice system 4. Invest in initiatives that promote educational and economic opportunities for Black and brown communities 5. Hold leadership accountable for the fair and equal treatment of all people, aligning with biblical teachings of justice and love 6. Emphasize that reparations are a mandate from God to rebuild relationships and create a better world 7. Encourage the Diocese of Maryland to collectively study, discuss, and take actionable steps towards reparations 8. Provide educational workshops and resources to facilitate understanding and commitment to reparative actions								
**The Movement for Black Lives (2016)**	M4BL Reparations Now Toolkit	The Movement for Black Lives is a decentralized coalition		People of African descent living in the U.S., particularly ADOS		The reparations plan encompasses a wide range of demands aimed at providing compensation, restoration, and systemic change for Black Americans			As of September 2024, the toolkit remains largely aspirational and serves as a comprehensive advocacy platform rather than an implemented policy
	**Demands to Institutional State, Federal, and International Governments as well as Organizational Actors** 1. Create federal and state legislation mandating the United States to recognize the enduring effects of slavery and develop and implement a plan to address these impacts 2. Provide free access and open admissions to public community colleges and universities, offering technical education in fields such as technology, trade, and agriculture, implementing educational support programs, retroactively forgiving student loans, and supporting lifelong learning initiatives 3. Provide reparations aimed at healing ongoing physical and mental trauma, while ensuring access to and control over food sources, housing, and land 4. Mandate public school curricula on the impacts of colonialism and slavery and fund the preservation and restoration of cultural assets and sacred sites to honor collective struggles and triumphs 5. Create mental health services, job programs, and other support programs for those affected by mass criminalization 6. Take action to address ongoing harms to Black people in the U.S., such as police violence, mass incarceration, deportation, segregation, employment and housing discrimination, and food apartheid								
**The National African American Reparations Commission (NAARC) (2015)**	10-Point Reparations Program	The NAARC is a decentralized coalition	All Black people who were harmed by systemic racism and oppression in America, particularly ADOS			The NAARC plan is a comprehensive proposal to address the historical and ongoing harms inflicted on Black people across a variety of domains			As of June 2025, the NAARC has been actively involved in promoting these ideas at local, state, and national levels. One key aspect of their current efforts is the push for the passage of H.R. 40 and local reparations initiatives
	**Demands to Local, State, and Federal Governments and Organizational Actors** 1. A formal apology from the US government, specifically the President and Congress, for their role in slavery and establish an African Holocaust Institute to educate the public​ 2. Provide resources for descendants of enslaved Africans to repatriate to African nations of their choice and support programs to bridge cultural gaps 3. Transfer of public lands from the federal government to the National Reparations Trust Authority to be used for the benefit of African Americans 4. Provide funds to support the development of Black-owned businesses and cooperative enterprises 5. Establish and fund health and wellness centers controlled by Black communities and support existing hospitals serving these communities​ 6. Invest in educational programs that empower Black communities and preserve their cultural heritage​ 7. Provide funds to ensure affordable housing for Black communities affected by systemic racism and historical injustices 8. Allocate funds to strengthen Black institutions, including historically Black colleges and universities 9. Support the preservation of historical sites and monuments significant to Black history​ 10. Address the harms caused by the criminal justice system, including mass incarceration and police violence								
**Vermont Racial Justice Alliance (2017)**	Burlington’s Racial Equity Strategic Roadmap	Burlington, VT			ADOS as well as other marginalized groups			Operation Phoenix includes restructuring public safety, implementing cultural empowerment programs, securing equal opportunities, and expanding racial equity, inclusion, and belonging	The Vermont Racial Justice Alliance has successfully led to Burlington’s declaration of racism as a public health crisis and the establishment of a reparations task force. The task force held one meeting in 2020, however, the current status is unclear
	**Preliminary Recommendations to the City and State Legislatures** 1. **Restructuring Public Safety**: Focus on ensuring everyone enjoys safety and protection under the law by restructuring the public safety apparatus in Burlington 2. **Implementing Cultural Empowerment**: Establish a Cultural Empowerment Community Collective to support communities of color through various programs and centers 3. **Securing Equal Opportunity**: Create an Office of Equal Opportunity to manage complaints and oversee new economic empowerment programs 4. **Expanding Racial Equity, Inclusion, and Belonging**: Fully fund and operationalize the Racial Equity, Inclusion, and Belonging (REIB) function to ensure comprehensive data analysis and policy implementation								

Some plans are preliminary, meaning that they are initial recommendations to stakeholders that would likely be modified after a reparations task force or overseeing bodies conducted further research. Certain plans (e.g., Vermont Racial Justice Alliance’s Operation Phoenix R.I.S.E., The Civil Justice and Restorative Justice Project’s Toolkit) include specific terminology to refer to reparative domains while others described their recommendations or demands more generally. Relevantly, the language around “recommendations” versus “demands” depends on language taken from the respective initiative. For example, the Black Panther Party frequently used “demand” in their initiative versus local or state taskforces who are making “recommendations” to other governing bodies

*Refers to plans that are extensive, wherein the overall recommendations are summarized

*ADOS;* American descendants of slavery
